# Origins, Dispersal, and Impact: Bidirectional Introgression Between Chinese and European Pig Populations

**DOI:** 10.1002/advs.202416573

**Published:** 2025-04-01

**Authors:** Yibin Qiu, Langqing Liu, Min Huang, Donglin Ruan, Rongrong Ding, Zebin Zhang, Enqin Zheng, Shiyuan Wang, Shaoxiong Deng, Xianglun Meng, Xinyan Cheng, Jiaxin Shi, Yingshan Yang, Fuchen Zhou, Sixiu Huang, Huaqiang Yang, Zicong Li, Gengyuan Cai, Zhenfang Wu, Jie Yang

**Affiliations:** ^1^ State Key Laboratory of Swine and Poultry Breeding Industry College of Animal Science and National Engineering Research Center for Breeding Swine Industry South China Agricultural University Guangzhou Guangdong 510642 China; ^2^ Guangdong Provincial Key Laboratory of Agro‐Animal Genomics and Molecular Breeding South China Agricultural University Guangzhou Guangdong 510642 China; ^3^ National and Regional Livestock Genebank Guangdong Gene Bank of Livestock and Poultry South China Agricultural University Guangzhou Guangdong 510642 China; ^4^ Guangdong Zhongxin Breeding Technology Co., Ltd Guangzhou Guangdong 511458 China; ^5^ Yunfu Subcenter of Guangdong Laboratory for Lingnan Modern Agriculture Yunfu Guangdong 527400 China

**Keywords:** ancient genome, bidirectional introgression, body size, pig, structural variation

## Abstract

Human mediated intra‐continental exchange of genetic material among domesticated organisms has never been restricted to a single direction. The introduction of pig breeds between China and Europe aims to enhance economically important traits in local populations. However, the reciprocal introgression pattern, specifically the role of introgressed genes and structural variations (SVs), remains underexplored. A global collection of whole‐genome resequencing data is utilized from 418 pigs to generate comprehensive dataset, including single‐nucleotide polymorphisms (SNPs) as well as SVs. The analysis reveals incomplete linkage disequilibrium between SVs and adjacent SNPs, highlighting the limitations of conventional SNP‐based analyses in capturing the genetic effects of SVs. By examining both population‐level SNPs and SVs, bidirectional introgression between Chinese and European pig populations is characterized. It is identified 3558 bidirectional introgressed genomic segments and 30 SVs, with haplotypes at *BMP2*, which are associated with improved body size. The origin and allele frequency trajectory of the *BMP2* segment are further validated using ancient genomes, suggesting that the body size‐enhancing haplotype likely originated from ancient European populations and has since maintained a relatively high allele frequency. Overall, the results highlight the significant role of bidirectional introgression in shaping the genetic composition and phenotypic traits in modern pig breeds.

## Graphical abstract

This study investigates bidirectional introgression between Chinese and European pig populations, revealing 3558 introgressed genomic segments and 30 structural variations. Analysis of the *BMP2* region suggests its role in body size enhancement. By integrating ancient and modern genomes, the study highlights the impact of introgression on genetic diversity and phenotypic traits in domesticated pigs.

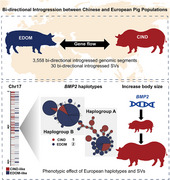

## 1 Introduction

Genetic introgression is a key evolutionary force that enhances genetic diversity and drives phenotypic evolution. Unlike the limited natural dispersal of wild fauna and flora, anthropogenic intervention has facilitated the rapid spread of domesticated organisms across continents, and importantly, this process is not restricted to one direction. Evidence of intra‐continental bidirectional migration has been observed in several domesticated species, including taurine/yak, llama/alpaca, sheep, goat, rice, and maize.^[^ ^1^, ^2^, ^3^, ^4^, ^5^, ^6^ ^]^ In pigs (*Sus scrofa*), independent domestication events of wild boars in China and Europe occurred ≈10 000 years ago.^[^ ^7^, ^8^, ^9^, ^10^ ^]^ The initial stages of breed formation were shaped by distinct breeding goals and partial geo‐graphic isolation, resulting in distinguishable phenotypic and genotypic differences between Chinese and European pigs. Subsequent hybridization and introgression between these two populations have led to the development of unique ancestral haplotypes within modern genomes. Historical records and genomic studies indicate that the intentional importation of Chinese pigs to Europe began in the late 18th and early 19th centuries, aimed at improving fertility in European commercial breeds like the Large White.^[^ ^11^ ^]^ More recently, Chinese breeders have integrated European commercial breeds into indigenous pig breeding programs to benefit from their superior growth rates, lean meat yield, and feed conversion efficiency.^[^ ^12^, ^13^, ^14^ ^]^ This integration is particularly evident in Northern Chinese indigenous breeds, including Laiwu, Min, and Lichahei pigs, which display significant hybridization with European commercial breeds.^[^ ^15^, ^16^, ^17^ ^]^

Previous population genetics studies based on single‐nucleotide polymorphism (SNP) data have primarily focused on one‐way introgression.^[^ ^11^, ^18^, ^19^ ^]^ However, the bidirectional introgression between Chinese and European pigs has not been thoroughly investigated. Moreover, quantifying the extent of introgressed genetic material and understanding its phenotypic effect remains an unanswered question to date. Structural variations (SVs) are recognized as an important mutational force shaping the genomic landscape during pig domestication.^[^ ^20^, ^21^ ^]^ Recent findings indicate a marked stratification of SVs between Chinese and European breeds, potentially associated with breed‐defining characteristics.^[^ ^22^ ^]^ The role and impact of introgressed SVs in the genetic exchange history between Chinese and European breeds merit further investigation through population‐based detection methods.

To address these gaps, we conducted a comprehensive assessment of the introgression landscape between Chinese and European pigs, utilizing a global dataset of 418 pig whole‐genome resequencing data. Our analysis specifically focuses on comparing genomic variations, including SVs, between Chinese and European pig populations to reveal patterns of bidirectional introgression. Finally, we determined the origins and potential contributions of introgressed haplotypes and SVs to economically important trait.

## 2 Results

### 2.1 Genetic Diversity, Population Structure, and Evidence of Introgression

**Figure 1:**
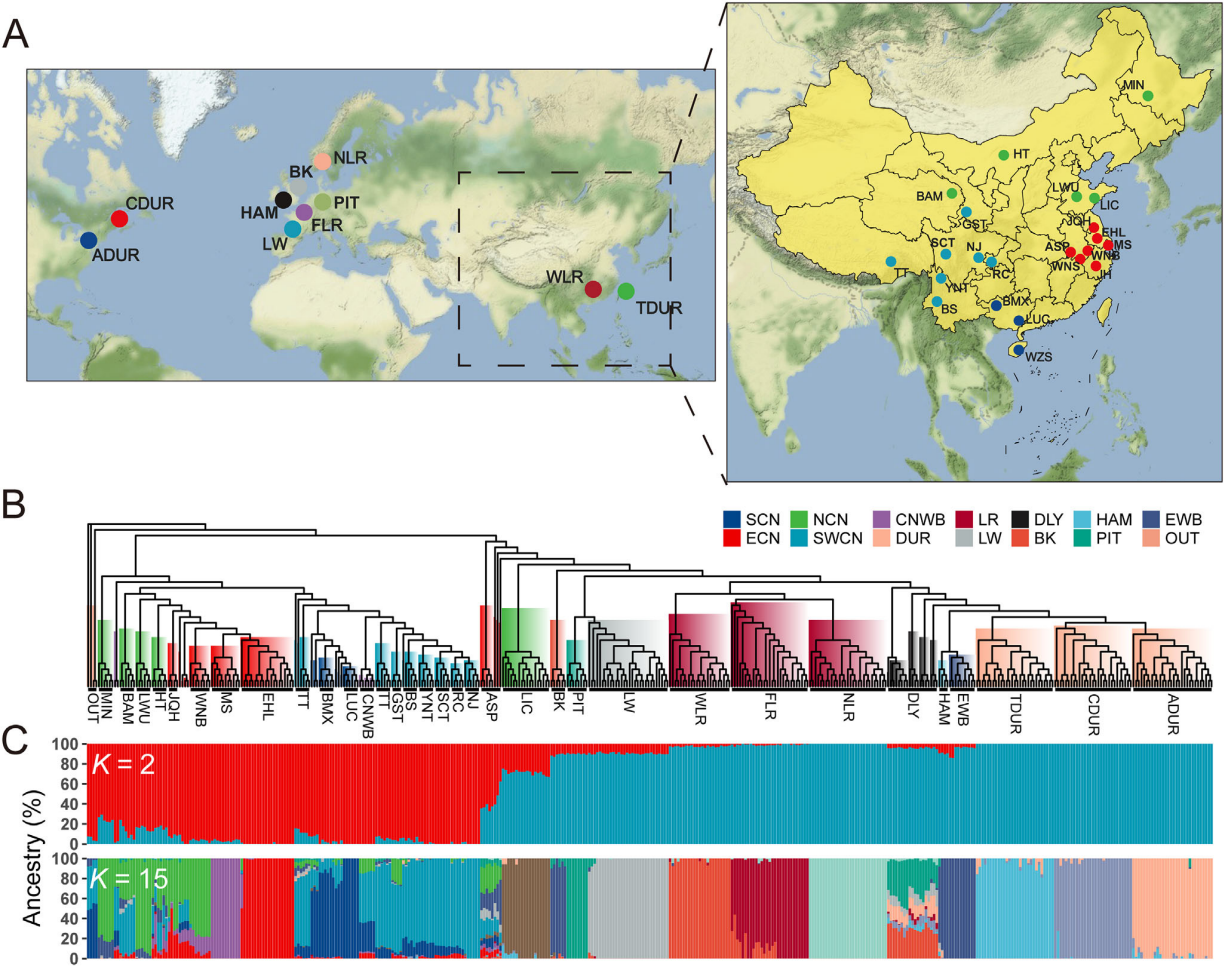
Phylogenetic relationships among Chinese and European pigs. A) Geographical location of pig populations sampled in this study. Each dot represents one population. Different colors denote different geographic groups. B) A neighbor‐joining tree of 418 pigs. Branch colors denote populations and geographic groups. C) Population structures were inferred using ADMIXTURE with the assuming number of ancestral cluster (*K* = 2 or 15). Each color represents one ancestral cluster and each vertical line represents one pig. The length of the colored segment in each vertical indicates the individual estimated fractional membership for each cluster. Abbreviations for these populations and their geographic groups are given in Table S1 (Supporting Information).

We estimated the demographic status of the Chinese and European pigs. Our analysis focused on 418 genomes representing a diverse array of populations: 22 Chinese indigenous populations (CIND), ten European domestic populations (EDOM), one Duroc × Landrace × Yorkshire population, one Chinese wild boar population (CNWB), one European wild boar population (EWB), and three outgroup species (**Figure** **1**A; and TableS1, Supporting Information).

We used the SNP dataset to construct a phylogenetic tree and estimate the lineage ancestry compositions between Chinese and European pigs (Figure 1B,C; and Figure S1, Supporting Information). The Chinese and European pigs form two distinct clades, which aligns with spatially restricted model of independent domestication.^[^ ^8^, ^23^, ^24^ ^]^ This differentiation is also revealed by pairwise genetic differentiation (*F*_ST_) analysis, linkage disequilibrium (LD), and the running of homozygosity (ROH) (Figures S2 and S3; Tables S2 and S3, Supporting Information). The substructure of Chinese and European populations was best captured with *K* = 15 (Figure 1C; and Figure S1, Supporting Information). Further, our Admixture and *D*‐statistics analyses detected the existence of gene flow signal between EDOM pigs and CIND pigs (Figure 1C; and Table S4, Supporting Information). We observed that the EDOM breeds, particularly Pietrain and Large White, shared a proportion of ancestry with Chinese populations in their genome (Figure 1C). In the opposite, the majority of Northern Chinese indigenous pigs, especially Lichahei pigs, are genetically closer to European pigs (Figure 1C; and Figure S4 and Table S4, Supporting Information). Taken together, the results showed a potential introgression signature in both directions between CIND and EDOM pigs.

### 2.2 SVs Distribution Reveals Population Structure and Extensive Introgression

**Figure 2:**
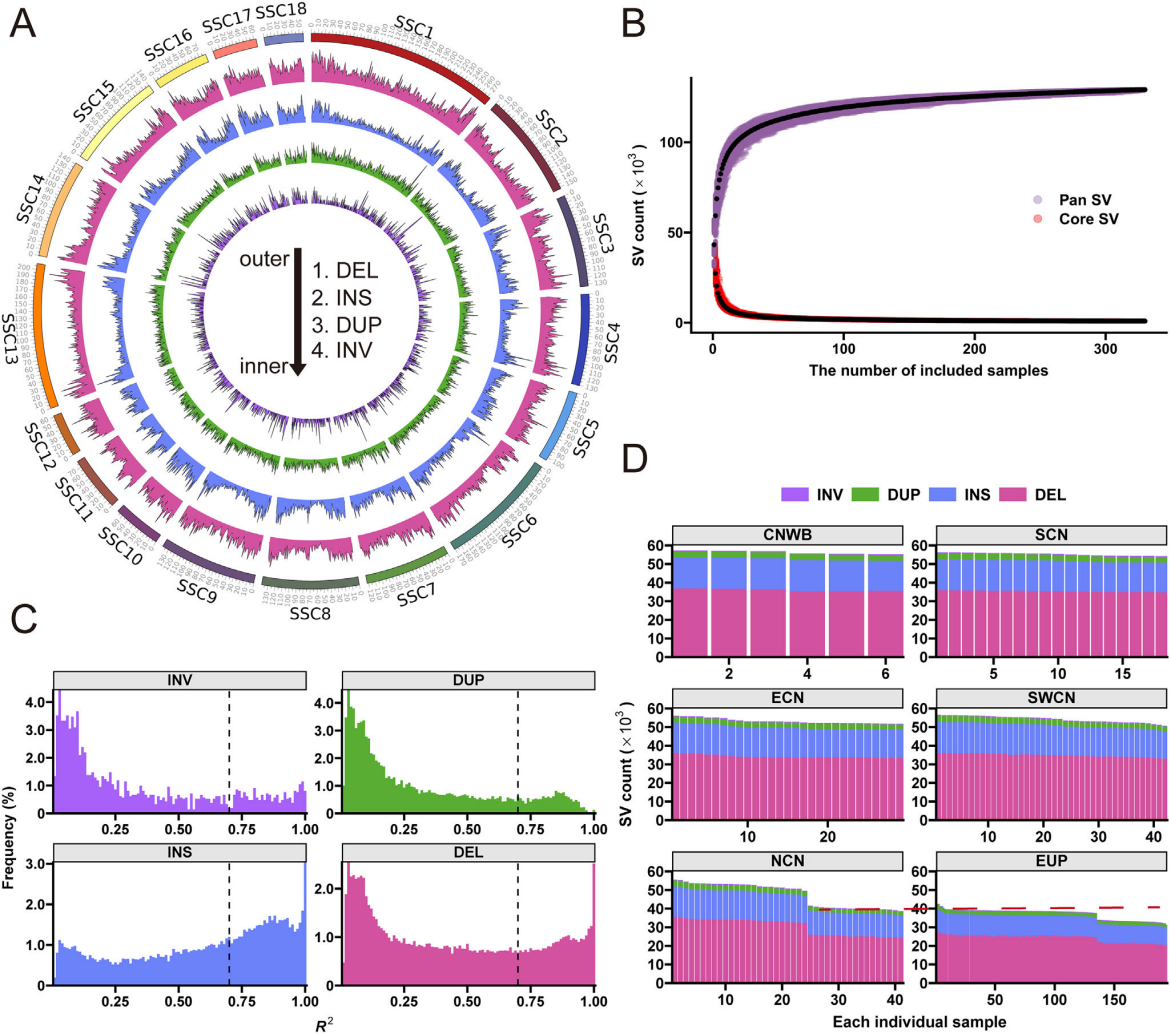
Characterization of structural variations (SVs) in Chinese and European pigs. A) The genome‐wide SV frequency distribution in 1 Mb nonoverlapping windows for 330 pigs. Circos from the outside (from 1 to 4) to the inside present deletion, insertion, duplication, and inversion, respectively. B) Pan‐SV and core‐SV counts with additional genomes. Black points correspond to mean value. C) The distribution of LD (*R* ^2^) between SVs and SNPs within 50 kb of the SVs. For each SV, the maximum *R* ^2^ with adjacent SNPs within 50 kb on either side is recorded. The dashed line indicates *R* ^2^ = 0.70. D) Stacked bar graph showing SV number and class from different geographic groups. Abbreviations for each group are given in Table S1 (Supporting Information).

To deeply survey the genomic landscape of SVs in CIND pigs and EDOM pigs, we performed SV genotyping for 330 pigs (see the Experimental Section). After filtering out low‐quality SVs, mainly translocations, we got a pan‐SV dataset with a total of 129 227 SVs, 79 358 deletions (DELs), 13 637 duplications (DUPs), 34 126 insertions (INSs), and 2106 inversions (INVs) (**Figure** **2**A; and Table S5, Supporting Information). Notably, the total number of discovered SVs did not substantially increase in 1000 iteratively random samplings when the individual count exceeded ≈300 (Figure 2B; and Table S6, Supporting Information). This suggests that our pan‐SV dataset efficiently captured the SV diversity throughout pig populations. DELs and INSs accounted for most of the SVs, and a large proportion (42.6%) of SVs had allele frequencies less than 0.1 (Figure S5A and Table S5, Supporting Information). We noted a sharp decrease in the frequency of INSs as their length increased (Figure S5B, Supporting Information), which might be because of the limitation of using short reads for detecting relatively large insertions.^[^ ^25^ ^]^ In‐depth annotation of the pan‐SV dataset showed that the majority of SVs were located in intergenic (49.0%) or intronic regions (39.5%), while only a small fraction (2.58%) of SVs were found overlapped with exons (Table S5, Supporting Information).

To explore the linkage between different classes of SVs and their surrounding SNPs, we calculated the maximum LD between SVs and adjacent SNPs within 50 kb flanking region (Figure 2C). Our analysis revealed that between 14.8% and 46.6% of the SVs were effectively tagged (with *R* ^2^ > 0.7) by adjacent SNPs across different SV classes. However, only small fractions (0.1%, 0.8%, 2.5%, and 3.0% for DUPs, INVs, DELs, and INSs, respectively) were in complete LD (*R* ^2^ = 1), indicating that these SVs may probably be neglected by traditional SNPs‐based analyses. To further reflecting the specific genetic structure within SVs, we used each class of SVs as genetic markers to assess population structure and compared these results with those obtained from SNP‐based structure inference. The first principal component (PC1) for all four SV classes distinctly separated the Chinese and Europeans pigs, mirroring the structure estimated from SNPs (Figures S4 and S6, Supporting Information). Interestingly, the second principal component (PC2) of DELs and INSs demonstrated separation between the EDOM breeds, while PC2 of DUPs and INVs showed the genetic difference among the four Chinese geographic populations. We subsequently constructed neighbor‐joining (NJ) tree and performed ADMIXTURE analyses based on SVs (Figure S7, Supporting Information). The results were consistent with those based on SNPs, showing the potential introgression signature observed in both directions. The similarity of SV distribution, particularly between Northern Chinese indigenous pigs and European pigs, strongly suggests an introgression history of SVs (Figure 2D; and Table S7, Supporting Information).

### 2.3 Genome‐Wide Bidirectional Introgression Patterns Between CIND and EDOM Pigs

**Figure 3:**
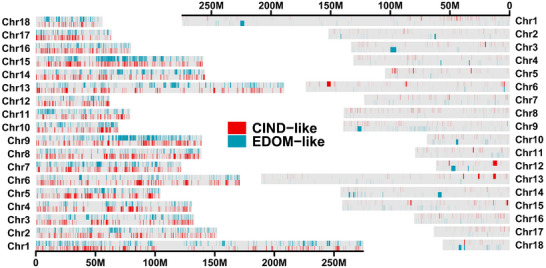
The genomic landscape of bidirectional introgression patterns between Chinese and European pigs. The left plot represents the genome‐wide distribution of introgressed fragments between Chinese and European pigs. The right plot represents the genome‐wide distribution of introgressed SVs between Chinese and European pigs.

As we found potential bidirectional introgression patterns between CIND and EDOM pigs with single‐nucleotide and structural variation markers, we performed a more comprehensive comparative analysis to infer genomic regions of interest. The introgressed segments were achieved through haplotype‐based relative identity by descent (rIBD) method^[^ ^11^ ^]^ and shared derived variants‐based *f* statistic (*f*_d_).^[^ ^26^ ^]^ *f*_d_ statistic was also used to further estimate the direction of gene flow (see the Experimental Section).^[^ ^26^ ^]^ In total, we detected 13 407 genomic fragments showing introgression signal from CIND pigs into EDOM pig population (**Figure** **3**; and Table S8, Supporting Information). In the opposite direction, from EDOM to CIND pigs, 17 071 putative introgressed fragments were detected (Figure 3; and Table S9, Supporting Information). The introgressed fragments classified as CIND‐like and EDOM‐like collectively cover 14.9% and 14.5% of the genome, respectively (Figure S8A, Supporting Information).

To supplement the above SNP‐based introgression results, we identified population‐specific SVs and tested for introgression using similar methods described in previous studies.^[^ ^27^, ^28^ ^]^ Our analyses yielded a total of 1613 CIND‐like and 1032 EDOM‐like introgressed SVs, spanning 0.95% and 1.39% of the genome, respectively (Figure 3; and Figure S8B; Tables S10 and S11, Supporting Information). By intersecting introgressed fragments and SVs between CIND and EDOM pigs in both direction, 3558 genomic fragments and 30 SVs showing bidirectional gene flow signals (Tables S12 and S13, Supporting Information). We performed functional annotation analysis on the genes overlapping the introgressed regions and those located near or overlapping SVs (Figure S9 and Table S14, Supporting Information). Our analysis suggested significant enrichment (corrected *P‐*value < 0.01) in various catalogs, including obesity‐related traits (e.g., *GMDS*, *PRKG1*, and *BMP2*), Height (e.g., *IGF2BP3*, *BMP2*, and *BMP3*), and Body mass index (e.g., *BMP2*).

### 2.4 Complex Introgression Pattern and Origin in *BMP2*

**Figure 4:**
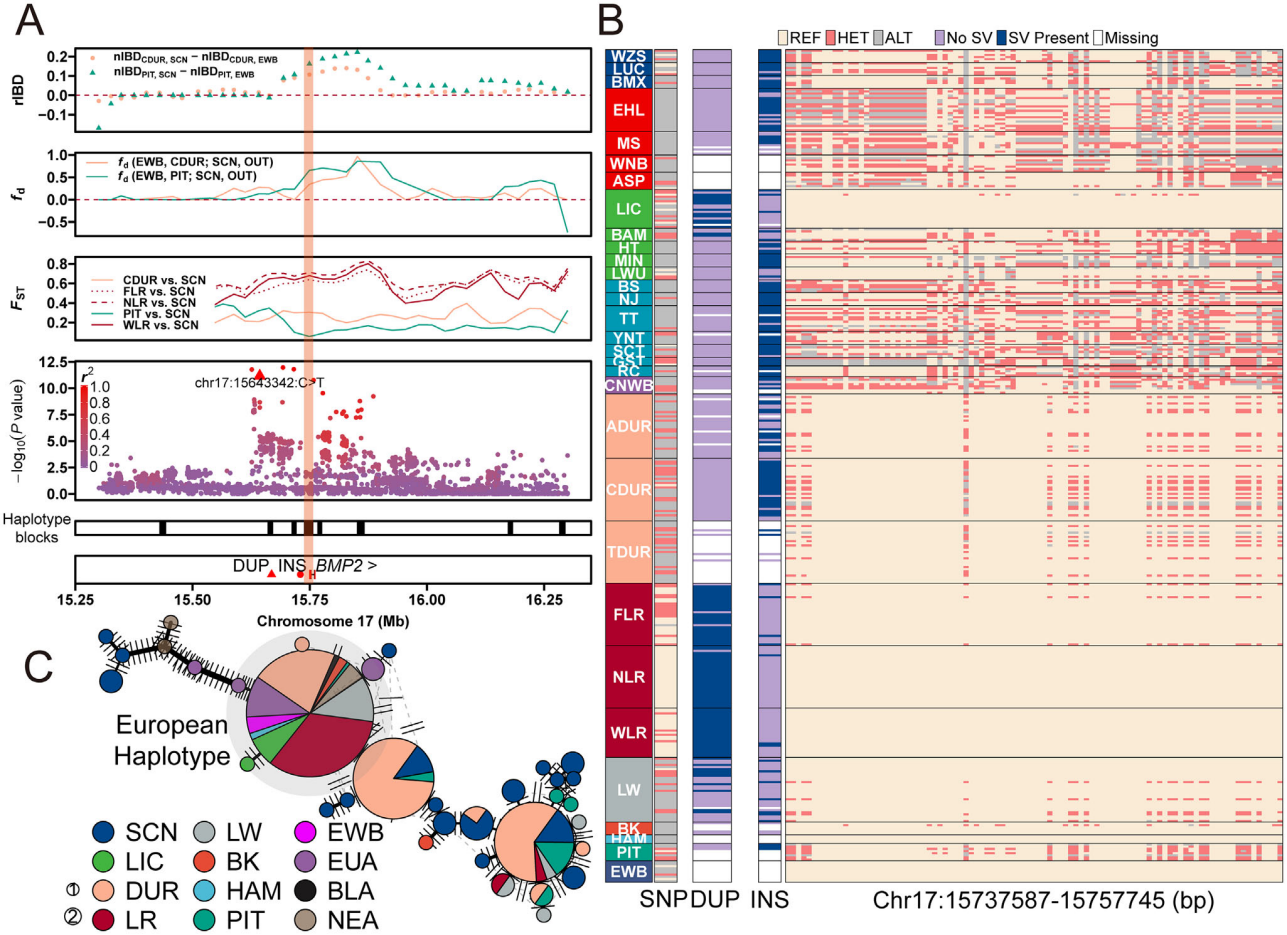
Identification and annotation of bidirectional introgression at the *BMP2* locus between Chinese indigenous pigs and European breeds. A) Distribution of rIBD (nIBD_EDOM, SCN_ − nIBD_EDOM, EWB_), *f*_d_ (((EWB, EDOM), SCN), Warthog), and *F*_ST_ surrounding the introgressed regions, respectively. Association within the 15.25–16.30 Mb region of chromosome 17, where the top hits in body height GWAS are located. Points indicate −log10(*P*‐value) along the chromosome using imputed genotypes. The significant SNP (chr17:15643342:C>T) was marked by triangle. *R* ^2^ between the SNPs and the top SNP are indicated by a gradient of color. Locations of LD blocks, genes, a duplication (chr17:15660659–15676598), and an insertion (chr17: 15731600) are indicated in the box below the plot and according to the Ensembl Release 110 annotation. The only gene with a symbol in this region is *BMP2*. B) Genotype patterns of the significant SNP (chr17:15643342:C>T), duplication (chr17:15660659–15676598), insertion (chr17:15731600), and *BMP2* haplotype (chr17:15737587–15757745). Each row represents an individual. C) The phylogenetic network of the European/Near Eastern *BMP2* haplotypes among modern and ancient pigs. Abbreviations for each population are given in Table S1 (Supporting Information).

Considering the well‐documented phenomenon of human‐mediated introgression, typically observed between specific pig breeds,^[^ ^11^, ^18^ ^]^ we selected one Northern Chinese indigenous breed known as Lichahei pigs, Southern Chinese indigenous pigs, and EDOM pigs to demonstrate the complex introgression patterns (**Figure** **4**A; and Figure S10; Tables S15 and S16, Supporting Information). Among the various genes identified in introgressed regions, we specifically highlight *BMP2* due to its strong association with body length and loin muscle depth in pigs.^[^ ^29^, ^30^, ^31^ ^]^ This gene is located within regions indicative of introgression between Southern Chinese indigenous pigs and Canadian Duroc or Pietrain (defined as regions where rIBD > 0). This was corroborated by two independent methods, *f*_d_ statistic and *F*_ST_ (Figure 4A; and Table S15, Supporting Information). In the meantime, we also detected a signal of European introgression in Lichahei pigs (Figure S10 and Table S16, Supporting Information).

Genotype comparisons for the SNPs in the introgressive regions were shown in Figure 4B; and Figure S11 (Supporting Information). Notably, we found that Duroc and Pietrain pigs displayed genotype patterns more closely resembling those of Southern Chinese indigenous pigs. In contrast, Lichahei pigs demonstrated genotype patterns more in line with those of EDOM pigs, such as Landrace, but from other Chinese indigenous pigs. Haplotype reconstruction revealed a 20.2 kb LD block (chr17:15737587–15757745) overlapping with *BMP2* gene (Figure 4A; and Figure S12, Supporting Information). Within this region, phylogenetic network analysis showed two major haplogroups (haplogroup A and B), representing Asian and European genetic component (Figure S13 and Table S17, Supporting Information). Within haplogroup A, haplotype I (hap I) was relatively prevalent among Southern Chinese indigenous pigs (13/36, 0.36). Additionally, we also found hap I present in EDOM breeds, particularly in Canadian Duroc (15/58, 0.26) and Pietrain (5/16, 0.31), suggesting that the Asian haplotypes are widespread and rarely fixed.^[^ ^11^ ^]^ Haplotype XXIX (hap XXIX) from haplogroup B was served as the major haplotype among EDOM pigs (334/434, 0.77), approaching fixation in Landrace pigs (159/162, 0.98). Notably, this haplotype was observed at a high frequency specifically in Lichahei pigs (34/36, 0.94), which was consistent with its European introgression history.^[^ ^17^ ^]^

Ancient DNA data can provide direct information on rising of haplotypes and changes in allele frequencies over time. To further investigate the origin and allele frequency trajectory of the *BMP2* haplotypes, we utilized 42 ancient pig genomes spanning from 12 000 to 1000 y before the present (BP) (Table S1, Supporting Information).^[^ ^32^ ^]^ A uniform manifold approximation and projection (UMAP) of the 20.2 kb LD blocks around the *BMP2* gene revealed seven clusters, including modern (Lichahei pigs, Southern Chinese indigenous pigs, European wild boars, and EDOM pigs) and ancient pigs (27 ancient European pigs, 12 ancient Near Eastern pigs, and three ancient Balkan pigs) (Figure S14, Supporting Information). It is noteworthy that a substantial proportion of the EDOM pigs and Lichahei pigs share a closer genetic affinity to the European wild boars and the ancient European/Near Eastern pigs. In contrast, the EDOM individuals harbored Asian haplotype are more closely related to the Southern Chinese indigenous pigs. The phylogenetic network analysis clearly showed the origin of the European/Near Eastern *BMP2* haplotypes (Figure 4C; and Table S18, Supporting Information). The majority of EDOM pigs (340/434, 0.78) and Lichahei pigs (35/36, 0.97) were clustered with the European wild haplotype (20/20, 1) and the ancient European/Near Eastern haplotype (78/84, 0.93), strongly indicating that the modern hap XXIX was likely derived from the ancient European populations and kept a relative high allele frequency afterward (Figure S15, Supporting Information).

In our previous body length genome‐wide association study (GWAS),^[^ ^30^ ^]^ we identified a high‐confidence candidate variant (chr17:15643342:C>T) located upstream of the *BMP2* gene. Given the high correlation coefficient between body length and body height (Figure S16, Supporting Information), we conducted a GWAS focused on body height in a population of 1618 Large White pigs (Figure 4A; and Figure S17, Supporting Information). We detected a highly significant peak located on chromosome 17, with the previously identified leading SNP (chr17:15643342:C>T) rounding out the top five significant SNPs in our GWAS results (Table S19, Supporting Information). We noted that the C allele, associated with increased body size, and haplotype XXIX were more prevalent in the Landrace breed compared to others (Figure 4B; and Figure S13; Tables S17 and S21, Supporting Information). Considering that this haplotype was nearly fixed in Landrace, we opted for the French Large White as our study group. We extracted 22 SNPs representing the *BMP2* haplotypes (chr17:15737587–15757745) in 1618 French Large White pigs to assess their association with both body length and body height (Table S20, Supporting Information). We discovered a statistically significant positive correlation between the European‐originated haplotype (hap XXIX) and body length (Wilcoxon rank sum test, *P* < 0.001) (Figure S18A, Supporting Information). Specifically, individuals carrying this haplotype were, on average, 3 cm longer than noncarriers. Additionally, we found that the homozygous carriers tended to have a 0.6 cm greater body height compared to noncarriers, although this difference did not achieve statistical significance in our analysis (Figure S18B, Supporting Information).

Within this introgressed genomic region, we identified a CIND‐like insertion (chr17:15731600) and an EDOM‐like duplication (chr17:15660659–15676598) located upstream of *BMP2* haplotypes (Figure 4A,B; and Table S21, Supporting Information). The insertion held a high‐frequency among Southern Chinese indigenous pigs (58.3%), Canadian Duroc (62.1%), and Pietrain (66.7%), but exhibited a low LD (*R* ^2^ = 0.11) with the significant SNP (chr17:15643342:C>T) (Figure 4B; and Tables S21 and S22, Supporting Information). Meanwhile, the duplication was largely restricted to Landrace and the Northern Chinese indigenous genome, with a frequency of 62.9% in Landrace and 38.2% in Lichahei pigs, but absent in other Chinese geographic populations (Figure 4B; and Table S21, Supporting Information). Moreover, the relatively high LD (*R* ^2^ = 0.56) indicated that this duplication was tagged to the significant SNP from body size (Table S22, Supporting Information). Given the relatively large size of this duplication (≈15.9 kb), we further validated its accuracy using long‐read sequencing data from 10 publicly available pig genomes (Figure S19 and Table S23, Supporting Information). SVs may impact the expression of nearby genes by changing the state of the cis‐regulatory elements.^[^ ^33^ ^]^ To infer the biological consequence of this duplication, we examined processed Chip‐seq data and ATAC‐seq data from Pan et al.^[^ ^34^ ^]^ Our analysis revealed that the duplication overlaps with H3 lysine 27 acetylation (H3K27ac) signals and open chromatin regions (Table S24, Supporting Information), highlighting its possible role in modulating gene expression by altering the cis‐regulatory elements.

## 3 Discussion

Despite growing evidence of introgression between Asian and European pigs, including Bosse et al.’s identification of Chinese‐derived haplotypes containing *AHR* that underwent positive selection for enhanced fertility in Large White populations,^[^ ^11^ ^]^ and Peng et al.’s report of an ≈2.65 Mb Chinese‐derived haplotype under selection in Duroc pigs,^[^ ^19^ ^]^ the extent and relative contributions of introgressed variants in pig domestication remained inadequately explored. A comprehensive catalog of variants from a diverse set of animals enabled us to uncover significant signals of genetic introgression between Chinese and European pig breeds, which were evident in both directions (Figures 2 and 3). This supported the hypothesis of bidirectional introgression during hybridization events and underscored the complexity of genetic exchanges in pig domestication.

While scenarios involving unidirectional gene flow are plausible, especially when the donor species is scarce, invasive, or migratory, recent evidence suggests that bidirectional gene flow was more prevalent.^[^ ^35^ ^]^ This phenomenon has been observed not only in pigs but also in other species, such as fish^[^ ^36^ ^]^ and mice,^[^ ^35^ ^]^ indicating that bidirectional gene flow may introduce functional novelties even among species with significant barriers. Our findings demonstrated that both Chinese and European pig populations have reciprocally contributed potentially functional genomic haplotypes, including SVs (Figure 3; and Tables S8–S14, Supporting Information). Many of these introgressed variants are implicated in growth‐related processes, suggesting that genetic exchanges may be linked to distinct breeding goals in China and Europe, each prioritizing different economical traits.

Our analysis revealed a novel introgressed region that encompassing the *BMP2* gene (Figure 4; and Figure S11, Supporting Information), demonstrating bidirectional introgression of haplotypes and SVs around the *BMP2* locus between European commercial pigs and Chinese indigenous breeds. Previous GWAS studies identified *BMP2* as a promising candidate gene associated with the body length and loin muscle depth in pigs.^[^ ^30^, ^31^ ^]^ Archaic pig genomes indicated that the haplotypes and SVs at the *BMP2* locus associated with longer body length originated in Europe. The introduction of those haplotypes from Europe to China, especially during the mid‐20th century through the admixture of the Lichahei breed with Duroc and Landrace, likely contributed to the larger body size observed in Lichahei pigs.^[^ ^17^ ^]^ While Chinese indigenous haplotypes were previously associated with enhanced disease resistance and reproductive performance^[^ ^18^ ^]^ (Figure S20, Supporting Information), determining the exact impact of introgressed haplotypes and SVs from these breeds on economically important traits remains challenging due to our limited understanding of genotype‐phenotype relationships (Supporting Information).

Furthermore, the extrapolation of the SV size indicated that our extensive SV dataset adequately captured SV diversity across pig populations (Figure 2B). However, the use of short‐read sequencing constrained the detection of complex SVs. Employing long‐read sequencing or multiplatform technologies,^[^ ^37^ ^]^ along with establishing a graph‐based pangenome that incorporates SVs,^[^ ^38^ ^]^ is crucial for a comprehensive exploration of the complete spectrum of structural variations in pigs. Although pangenome‐based introgression analyses remain unexplored in pigs, similar approaches have proven effective in cattle for disentangling adaptive introgression events.^[^ ^28^ ^]^ The implementation of these approaches in pig populations could further elucidate the role of genetic exchanges in shaping economically important traits.

## 4 Conclusion

Collectively, we elucidate the genomic bases of bidirectional introgression in pigs resulting from intra‐continental trade between China and Europe. Our findings demonstrate that both Chinese and European pig populations have reciprocally contributed functional genomic haplotypes, including SVs. With a representative case of bidirectional introgression at the *BMP2* locus, we show the widespread dispersal of Chinese haplotypes and SVs across European pig breeds, and the potential phenotypic effect of European haplotypes and SVs on commercial traits in Chinese breeds. Overall, our study highlights the profound impact of reciprocal gene flow in shaping the genetic and phenotypic constitution of modern pig breeds that we recognize today. It reveals the complex patterns of genetic exchange and lays a foundational basis for future genomic investigations in this area.

## 5 Experimental Section

### WGS Data Collection

Genome‐wide resequencing data were obtained from diverse sources, including 129 pigs newly reported in this study, along with 88 pigs retrieved from the prior study.^[^ ^30^ ^]^ Among them, 30 American Duroc (ADUR), 29 Canadian Duroc (CDUR), 29 Taiwan Duroc (TDUR), 30 French Large White (LW), 23 Wen's Landrace (WLR), 29 French Landrace (FLR), 29 Norwegian Landrace (NLR), and 18 Lichahei pigs (LIC), all sourced from Wen's Food Group Co., Ltd. (Yunfu, Guangdong, China) were included. Additionally, sequences for 201 animals were downloaded from the Sequence Read Archive (Table S1, Supporting Information). In total, 418 samples were utilized in this study, including European domestic pigs (EDOM, *n* = 217), Duroc × Landrace × Yorkshire pigs (DLY, *n* = 19), European wild boar (EWB, *n* = 10), Chinese indigenous pigs (CIND, *n* = 160), Chinese wild boar (CNWB, *n* = 8), and three outgroup species (Sumatran wild boars, *n* = 2; *Sus barbatus*, *n* = 1; *Phacochoerus africanus*, *n* = 1).

Within the Chinese indigenous pigs, the pigs were further classified based on their geographical origins, as proposed in previous studies,^[^ ^19^, ^39^ ^]^ resulting in the following subgroups: Southern Chinese (SCN, *n* = 18), Eastern Chinese (ECN, *n* = 55), Northern Chinese (NCN, *n* = 42), and Southwestern Chinese indigenous pigs (SWCN, *n* = 45). Table S1 (Supporting Information) provides the detailed information of the samples, including the sequencing platforms, accession numbers, sample sizes, and average sequencing coverage.

### Read Mapping and SNP Calling

Raw reads were processed by fastp v0.23.2^[^ ^40^ ^]^ with default parameters to remove the adapters and low‐quality sequences. Clean reads from each individual were aligned to the pig reference genome (Sscrofa 11.1) by the mem algorithm from BWA v0.7.17.^[^ ^41^ ^]^ SAMTools v1.3.1,^[^ ^42^ ^]^ and Picard v2.7.1 (http://broadinstitute.github.io/picard/) were used for data sorting and duplicate marking, respectively. Variant calling of SNPs was performed using GATK v4.1.8.1.^[^ ^43^ ^]^ Hard filtering was applied on SNPs under the criteria of QD < 2.0 || FS > 60.0 || MQ < 40.0 || MQRankSum < −12.5 || ReadPosRankSum < −8.0 || SOR > 3.0 via the VariantFiltration function, as recommended by GATK's best practices. Finally, variants with minor allele frequency (MAF) < 0.05 or call rate < 90% were removed using a combination of VCFtools v0.1.13^[^ ^44^ ^]^ and BCFtools v1.15.1,^[^ ^42^ ^]^ resulting in 15 771 704 bi‐allelic SNPs.

### Structural Variant Calling

Structural variant calling and genotyping was performed using Manta v1.6 ^[^ ^45^ ^]^ + GraphTyper v2.7.5,^[^ ^46^ ^]^ as it shown a good performance in recent studies.^[^ ^47^, ^48^, ^49^ ^]^ Manta v1.6 was run^[^ ^45^ ^]^ on the 332 pigs which have sequence depth ≥ 10 and genomic coverage ≥ 93% to generate individual VCFs for each sample. Then variants were extracted that “PASS” all the quality thresholds of Manta using vcffilter from Vcflib v1.0.3.^[^ ^50^ ^]^ ConvertInversion.py was subsequently run provided by Manta to reformat inversions into single inverted sequence junctions.^[^ ^49^ ^]^ The svimmer v0.1 (available at https://github.com/DecodeGenetics/svimmer) was used to merge SVs in all 332 samples under default setting. The merged dataset comprised 280 312 SVs. The merged dataset across all samples (*n* = 332) was subsequently genotyped using Graphtyper v2.7.5.^[^ ^46^ ^]^ Deletions, insertions, duplications, and translocations detected by the “aggregate” model and inversions detected by the “breakpoint” model, as suggested by Eggertsson et al. were extracted.^[^ ^46^ ^]^ The SVs with sizes over 5 Mb were excluded. Individuals with SV call rate < 0.9 were removed. The final SV dataset included 330 individuals with 79 358 DELs, 34 126 INSs, 13 637 DUPs, 2106 INVs, and 17 065 translocations.

### Functional Annotation of SVs

The ANNOVAR software was used^[^ ^51^ ^]^ to annotate the genomic features of the identified SVs. The SVs were classified into 13 groups: downstream, exonic, intergenic, intronic, ncRNA exonic, ncRNA intronic, ncRNA splicing, splicing, upstream, and untranslated region (UTR: UTR3 and UTR5).

### Genetic Diversity Statistics

To evaluate genetic diversity, a SNP dataset was utilized comprising 14 779 826 autosomal SNPs. Nucleotide diversity (π) was calculated using VCFtools v0.1.13.^[^ ^44^ ^]^ PLINK v1.90^[^ ^52^ ^]^ was used to calculated the proportion of polymorphic markers (*P*_N_), observed heterozygosity (*H*_o_), expected heterozygosity (*H*_e_), ROH, and LD for all SNP pairs, following the approach used in previous studies.^[^ ^53^, ^54^ ^]^

### Population Structure and Phylogenetic Analysis

*F*_ST_ per population was computed using VCFtools v0.1.13,^[^ ^44^ ^]^ and visualized using the ComplexHeatmaps R package.^[^ ^55^ ^]^ Principal component analysis (PCA) was conducted using GCTA v1.94.0.^[^ ^56^ ^]^ LD was calculated for SNP pairs within a 500 kb window using PopLDdecay v3.42.^[^ ^57^ ^]^ A NJ tree with 100 bootstraps was constructed using VCF2Dis v1.50 (available at https://github.com/BGI‐shenzhen/VCF2Dis). The phylogeny was visualized using the ggtree R package.^[^ ^58^ ^]^ Admixture analysis was performed using ADMIXTURE v1.3.0^[^ ^59^ ^]^ with the cluster number *K* ranging from 2 to 20. Prior to the admixture analysis, the SNP dataset with 418 samples was pruned using the ′–indep‐pairwise 50 10 0.3″ command in PLINK v1.9,^[^ ^52^ ^]^ resulting in 966 327 SNPs included in this analysis. Ten random seeds were run for each *K*, and the seed with the lowest cross‐validation error was selected (Table S25, Supporting Information).

For the SV dataset, separate PCA analyses for each SV class (DEL, DUP, INS, and INV) were performed. NJ trees and admixture analyses were conducted using all SV classes (Table S26, Supporting Information). The software parameter settings were consistent with the SNP‐based analysis.

### Gene Flow Among Populations

To detect potential gene flow between CIND and EDOM pigs, the Patterson's *D* and f4‐ratio statistics using the DtriosParallel script in the Dsuite software was conducted.^[^ ^60^ ^]^ The *D*‐value was evaluated using a two‐tailed *Z*‐test, with |*Z*‐score| > 3 considered significant.^[^ ^61^ ^]^

### Identification of Introgressed Genomic Regions

Identity was employed by descent (IBD) sharing approach^[^ ^11^ ^]^ combined with *f*_d_ statistic^[^ ^26^ ^]^ to further localize the introgressed genomic regions across the entire genome.

For the IBD sharing approach, BEAGLE v4.1 was used^[^ ^62^ ^]^ to conduct 10 independent cycles of phasing and pairwise IBD detection. Then the identified IBD tracts were merged based on the Beagle probability scores.^[^ ^62^ ^]^ The relative IBD (rIBD) was calculated for each 50 kb window (25 kb sliding) using the following formula

(1) $$rIBD=nIBD_{r,d}-nIBD_{r,b}$$

where *nIBD*_*r*,*d*_ represents the normalized IBD (nIBD) value between the recipient‐donor pair, and *nIBD*_*r*,*b*_ represents the nIBD value between the recipient‐background pair. The nIBD value was obtained by the formula *nIBD*  =  *cIBD*/*tIBD*, where cIBD indicates the count of haplotypes IBD between two groups, and tIBD represents the total pairwise comparisons between two groups. The range of nIBD values varied between 0 (indicating no IBD detected) and 1 (indicating all individuals in the group share IBD). The threshold for extreme IBD to be considered as putative introgression windows was set to top 1% of rIBD values.^[^ ^18^, ^54^ ^]^

For each putative introgression windows, the ABBABABAwindows.py^[^ ^26^ ^]^ was used to calculate Patterson's *D* and *f*_d_ statistics simultaneously. The *f*_d_ values were adjusted to 0 at windows where *D* values are negative. Different combinations of populations were tested, leading to the calculation of the following arrangements

- Arrangement 1: *D* (EWB, recipient; donor, outgroup)

The European wild boar (EWB) served as the background population, while each population from the European domestic pigs functioned as the recipient. The potential donor pigs, which was Southern, Eastern, Northern, and Southwestern Chinese indigenous pigs, one at a time. Warthog was chosen as the sole outgroup for ancestral allele inference.^[^ ^8^ ^]^

ii. Arrangement 2: *D* (CNWB, recipient; donor, outgroup)

The Chinese wild boar (CNWB) was selected as the background population. The recipient pigs, representing Southern, Eastern, Northern, and Southwestern Chinese indigenous pigs, were tested against potential donor pigs, encompassing each population from the European domestic pigs. Warthog was chosen as the sole outgroup for ancestral allele inference.

Additionally, a comparative analysis of the *f*_d_ values for each window was conducted to predict the potential directions of introgression.^[^ ^4^, ^26^ ^]^ For instance, assuming gene flow from CIND into EDOM pigs as the true direction, arrangement 1 would yield a reliable estimate, while arrangement 2 would likely provide an underestimate (Tables S8 and S9, Supporting Information).

### Identification of Population‐Specific and Introgressed SVs

A search was conducted for candidate population‐specific and introgressed SVs in Chinese and European breeds using methods similar to those described in prior studies.^[^ ^27^, ^28^ ^]^ For example, the *F*_ST_ was calculated for SVs by comparing Northern Chinese indigenous pigs with other Chinese indigenous pigs, using VCFtools v0.1.13.^[^ ^44^ ^]^ Subsequently, 1000 permutation tests were conducted to obtain empirical *P*‐values. SVs were determined as population‐ specific in the Northern Chinese indigenous pigs that satisfy (1) *F*_ST_ value > top 5%, (2) empirical *P*‐value < 0.05, and (3) maximum missingness rate < 0.1 and MAF > 0.01. The candidate introgressed SVs were determined, if the population‐stratified SVs were flanked by inferred introgression regions (see above).

### Validation of the 15.9 kb Duplication with Long‐Read Sequencing Data

To validation the 15.9 kb duplication (chr17:15660659–15676598), Oxford Nanopore Technologies (ONT) sequencing data from 10 genomes (PRJCA005901) were utilized.^[^ ^21^ ^]^ The sequencing reads were mapped to the pig reference genome (Sscrofa 11.1) using minimap2 v2.28,^[^ ^63^ ^]^ and SV were called using sniffiles2 v2.5.3 with default parameters.^[^ ^64^ ^]^ The 15.9 kb duplication was visualized using Integrative Genomics Viewer v2.18.4 software.^[^ ^65^ ^]^

### Ancient DNA Data Processing

A total of 42 ancient genomes were downloaded from the Sequence Read Archive (Table S1, Supporting Information).^[^ ^21^ ^]^ Data processing was performed using the nf‐core/eager v2.5 pipeline.^[^ ^66^ ^]^ Briefly, raw sequencing reads were subjected to quality filtering using fastp v0.23.2. Cleaned reads were aligned to the reference genome (Sscrofa 11.1) using BWA v0.7.17 (“aln” and “samse” commands). Postalignment processing, including sorting and duplicate marking, was conducted using SAMTools v1.3.1 and Picard v2.7.1 (http://broadinstitute.github.io/picard/). Molecular damage patterns were assessed using MapDamage v2.2.2.^[^ ^67^ ^]^ Finally, variant calling was performed using the GATK v4.1.8.1 HaplotypeCaller program with default parameters.

### Analysis of *BMP2* Haplotype Pattern, Network, and Origin

Phased SNPs were extracted within a 100 kb flanking region around the *BMP2* gene from 418 individuals and visualized specific genotypes pattern in a heatmap (Figure S11, Supporting Information). Three pig populations (Southern Chinese indigenous, Lichahei, and European domestic pigs) were focused on for haplotype estimation using PLINK v1.90, resulting in 235 samples and 22 SNPs (MAF > 0.05) in a 20.2 kb *BMP2* haplotype (Figure S12, Supporting Information). The haplotype network was constructed using the R package PEGAS.^[^ ^68^ ^]^

To further visualize the origin and allele frequency trajectory of the *BMP2* haplotype, ancient genomes were incorporated. Given the low coverage of ancient DNA data,^[^ ^32^ ^]^ variants were filtered with a call rate < 100% using VCFtools v0.1.13, retaining only shared markers between the modern pig populations (without applying MAF filtering). Genotypes from ancient and modern populations were merged using BCFtools v1.15.1, resulting in 38 SNPs within the 20.2 kb *BMP2* haplotype. PCA was conducted using GCTA v1.94.0^[^ ^56^ ^]^ in these SNPs. Then a UMAP was run on the top five PCs that show population structure between ancient genomes and three pig populations. This was implemented using the R package uwot v0.1.16 (https://CRAN.R‐project.org/package=uwot), with the following settings: initialization for the coordinated as “spca,” min_dist = 1.5, and n_neighbors = 5. The haplotype network was also constructed using the R package PEGAS.^[^ ^68^ ^]^

### Genome‐Wide Association Study

A total of 1618 French Large White pigs were genotyped using the Affymetrix PorcineWens55K SNP chip (Affymetrix, Santa Clara, CA) in the previous study.^[^ ^30^ ^]^ Body heights of all pigs were measured using a meter ruler when they reached ≈100 kg (100 ± 5 kg) body weight. The SNP chip data were imputed to a whole genome sequence level using the Swine IMputation platform.^[^ ^30^ ^]^ Variants with MAF < 0.01 and Hardy–Weinberg *P*‐value < 10^−6^ were eliminated. After filtering, a final set of 17 000 021 variants remained for the GWAS. For the GWAS of body height, GEMMA software v0.98.1 was employed,^[^ ^69^ ^]^ utilizing a univariate mixed linear model (MLM). The MLM can be represented as follows

(2) y=Wα+Xβ+u+ε

where y represents a vector of the phenotypes in the LW pigs; W is the incidence matrix of covariates, including fixed effects of year‐season, and the top five eigenvectors obtained prior to this analysis using the GCTA software v1.92.4beta;^[^ ^56^ ^]^ α represents the vector of corresponding coefficients including the intercept; X is the vector of variant genotypes; β specifies the corresponding effect size of the marker; u is the vector of random effects, with u ~ MVN_n_ (0, λτ^− 1^K); ε is the vector of random residuals, with ε ~ MVN_n_ (0, τ^− 1^I_n_); λ is the ratio between the two variance components;  τ^− 1^ is the variance of the residual errors; K is a genomic relatedness matrix estimated by Affymetrix Wens 55K SNP chip; I_n_ is an n × n identity matrix; MVN_n_ denotes the n‐dimensional multivariate normal distribution. A genome‐wide significance threshold of *P* = 5 × 10^−8^ was used to declare significance.^[^ ^70^ ^]^

### Functional Enrichment Analysis

The genes overlapping with the concatenated introgressed regions and those located near or overlapping SVs were subjected to annotation. Pig Ensembl gene IDs were converted to orthologous human Ensembl gene IDs using BioMart (http://asia.ensembl.org/biomart/martview). The National Human Genome Research Institute (NHGRI) GWAS catalog annotation was conducted using KOBAS v3.0.^[^ ^71^ ^]^ Benjamini–Hochberg^[^ ^72^ ^]^ adjusted *P*‐values were estimated with *P*‐value < 0.01 as statistically significant.

### Ethical Statement

All experimental protocols were approved by the Animal Care and Use Committee of the South China Agricultural University (approval number: SYXK 2019‐0136, Guangzhou, China). No anesthesia or euthanasia was performed on the experimental animals in this study.

## Conflict of Interest

The authors declare that they have no known competing financial interests or personal relationships that could have appeared to influence the work reported in this paper.

## Author Contributions

Y.Q. and L.L. contributed equally to this work. Z.W., J.Y., and L.L. conceived and designed the experiment. Y.Q., M.H., D.R., R.D., Z.Z., E.Z., S.W., S.D., X.M., X.C., J.S., Y.Y., F.Z., S.H., H.Y., Z.L., and G.C. collected the samples, recorded the phenotypes, and performed the experiments. Y.Q. analyzed the data. Y.Q. and L.L. wrote the manuscript. Z.W., L.L., and J.Y. improved the manuscript. Z.W. contributed to the materials. All authors reviewed the manuscript.

## Supporting information



Supporting Information

Supplemental Tables

## Data Availability

The data that support the findings of this study are available from the corresponding author upon reasonable request.
