# Supplementary material for: Origins, Dispersal, and Impact: Bidirectional Introgression Between Chinese and European Pig Populations
Source: Adv Sci (Weinh). 2025 Apr 1;12(22):2416573. doi: 10.1002/advs.202416573 (PMC12165087; doi:10.1002/advs.202416573)
Supplement: Supplementary file 1 — Supporting Information [file ADVS-12-2416573-s001.docx]

**Supporting Information for**

**Origins, Dispersal, and Impact:** **Bidirectional Introgression between Chinese and European Pig Populations**

Yibin Qiu^1,4†^, Langqing Liu^1,2,4*†^, Min Huang^1,4^, Donglin Ruan^1,4^, Rongrong Ding^1,3,4^, Zebin Zhang^1,2,4^, Enqin Zheng^1,2,4^, Shiyuan Wang^1,4^, Shaoxiong Deng^1,4^, Xianglun Meng^1,4^, Xinyan Cheng^1,4^, Jiaxin Shi^1,4^, Yingshan Yang^1,4^, Fuchen Zhou^1,4^, Sixiu Huang^1,2,4^, Huaqiang Yang^1,3,4^, Zicong Li^1,2,4^, Gengyuan Cai^1,2,3^, Zhenfang Wu^1,2,5*^ and Jie Yang^1,2,4*^

^1^State Key Laboratory of Swine and Poultry Breeding Industry, College of Animal Science and National Engineering Research Center for Breeding Swine Industry, South China Agricultural University, Guangzhou, Guangdong, China

^2^National and Regional Livestock Genebank, Guangdong Gene Bank of Livestock and Poultry, South China Agricultural University, Guangzhou, Guangdong, China

^3^Guangdong Zhongxin Breeding Technology Co., Ltd, Guangzhou, Guangdong, China

^4^Guangdong Provincial Key Laboratory of Agro-animal Genomics and Molecular Breeding, South China Agricultural University, Guangzhou, Guangdong, China

^5^Yunfu Subcenter of Guangdong Laboratory for Lingnan Modern Agriculture, Yunfu, Guangdong, China

^†^ These authors contributed equally to this work.

^*^ Corresponding authors: langqing.liu@scau.edu.cn; wzfemail@163.com; jieyang2012@hotmail.com

**This word file includes:**

Introgression of Chinese Haplotypes and Associated SVs in Large White Pigs

Supplementary Figure S1 to S20

Legends for Supplementary Table S1 to S26

**Other supporting materials for this manuscript include the following:**

Supplementary Table S1 to S26

**Introgression of Chinese Haplotypes and Associated SVs in Large White Pigs**

In 2019, Chen et al. ^[1]^ identified Southern Chinese-derived haplotypes containing the *GOLM1-NAA35* locus that were introgressed into European pigs and subsequently underwent selection to enhance disease resistance during the development of French Large White (FLW) pigs. Additionally, an introgressed *KATNAL1* haplotype from Eastern Chinese pigs was preferentially selected to improve fertility in FLW boars. In this study, we performed a haplotype-based relative identity by descent (rIBD) between 160 Chinese indigenous pigs and 30 FLW pigs at these two loci. Our results confirmed the presence of Southern Chinese-derived haplotypes in FLW pigs, with a strong introgression signal at the *GOLM1-NAA35* locus (Table S8; Figure S20A). Similarly, we detected Eastern Chinese-derived haplotypes in FLW pigs, exhibiting a strong introgression signal at the *KATNAL1* locus (Table S8; Figure S20C). Within these introgressed genomic regions, we identified four CIND-like structural variants (SVs) located upstream of each locus (Table S10; Figure S20 C and D). Furthermore, we detected a 357 bp deletion (DEL1; chr11:6,960,754-6961,111) and a 51 bp insertion (INS2; chr11:6,962,471) upstream of *KATNAL1*, both of which overlap with previously reported potential regulatory elements (Table S24) ^[2]^. This suggests that these structural variants may influence gene expression by affecting cis-regulatory mechanisms.

**Reference**:

[1] H. Chen, M. Huang, B. Yang, Z. Wu, Z. Deng, Y. Hou, J. Ren, L. Huang, *GigaScience* **2020**, *9*, giaa014.

[2] Z. Pan, Y. Yao, Hongwei Yin, Z. Cai, Y. Wang, L. Bai, C. Kern, M. Halstead, G. Chanthavixay, N. Trakooljul, K. Wimmers, G. Sahana, G. Su, M. S. Lund, M. Fredholm, P. Karlskov-Mortensen, C. W. Ernst, P. Ross, C. K. Tuggle, L. Fang, H. Zhou, *Nat. Commun.* **2021**, *12*, 5848.

**Supplementary Figure S1 to S20**

**
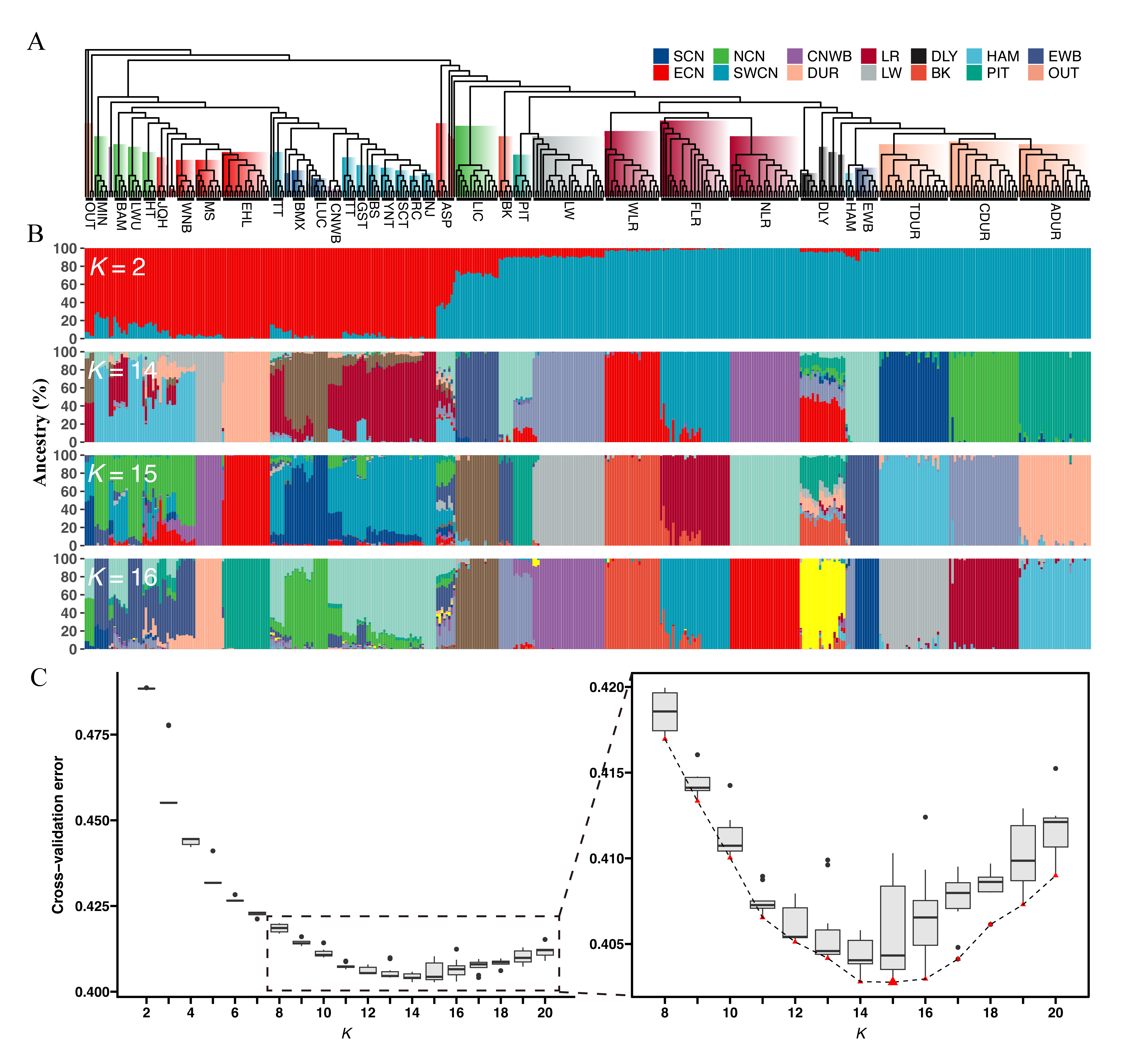
**

**Figure S1. Phylogenetic relationships and population structure of Chinese and European pigs based on SNP.** (A) A neighbor-joining tree based on SNPs. Branch colors denote populations and geographic groups. Abbreviations for each population are given in Supplementary Table S1. (B) Admixture analysis based on SNPs. (C) Cross-validation errors for each *K* and those for *K* from 8 to 20 are shown in the right panel. Each box plot represents the Cross-validation errors from 10 independent runs with randomly chosen seeds.

**
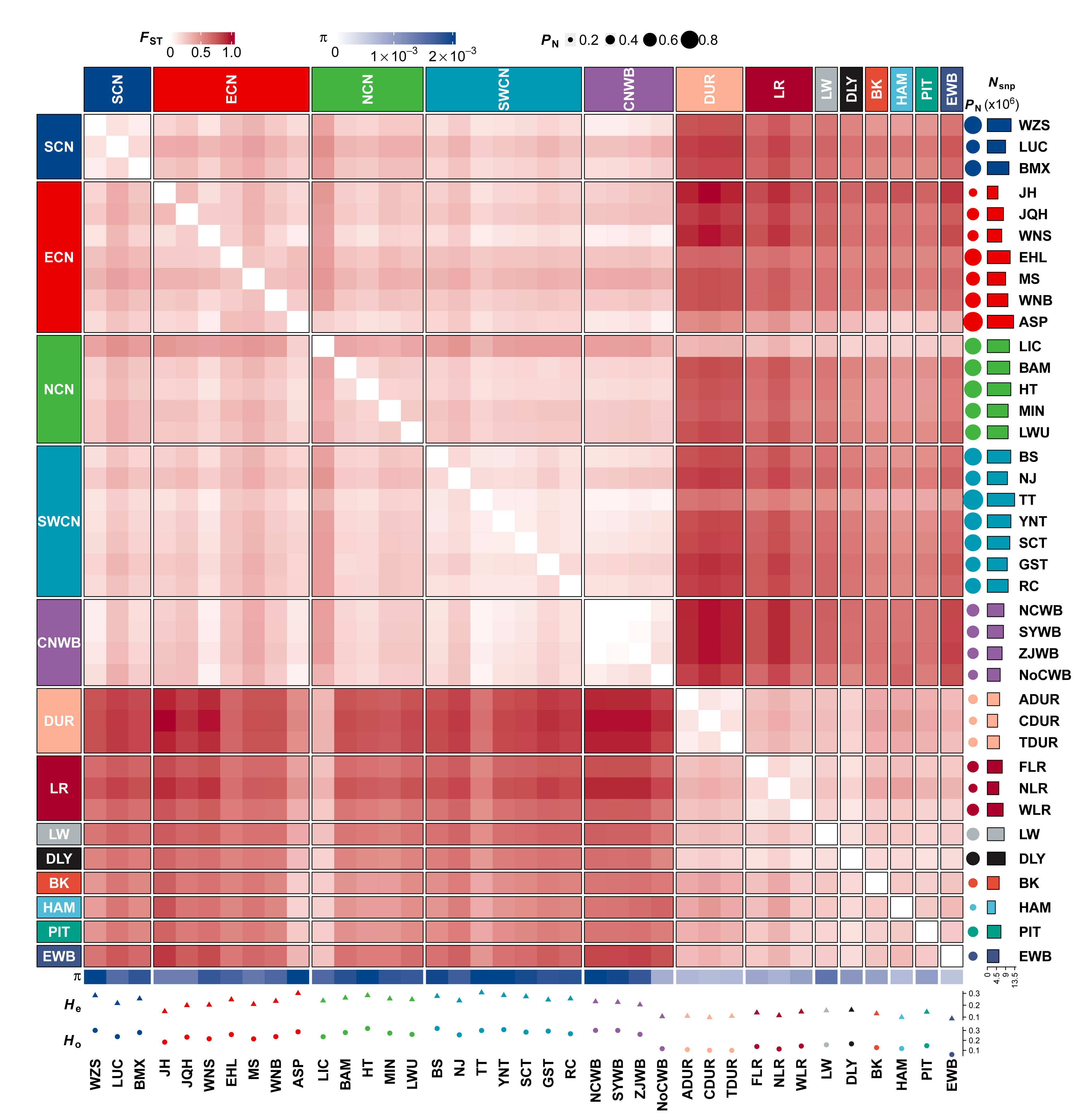
**

**Figure S2. Genomic diversity and pairwise genetic differentiation (*F*_ST_) per population.** *N*_snp_, the number of qualified SNPs after filtering process. *P*_N_, the proportion of polymorphic markers. *H*_o_, observed heterozygosity. *H*_e_, expected heterozygosity. 𝜋, Nucleotide diversity. Abbreviations for each population are given in Supplementary Table S1.

**
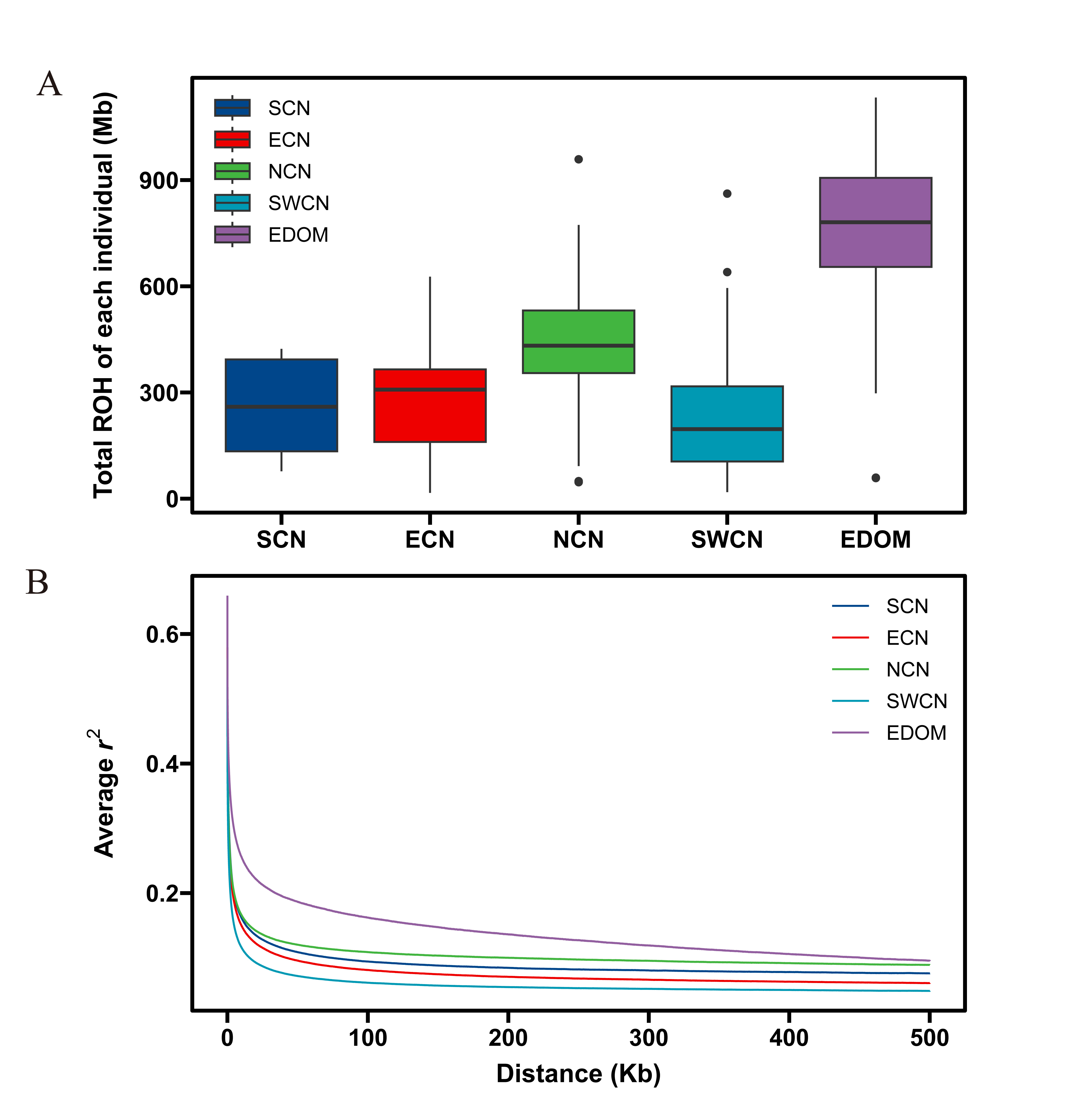
**

**Figure S3. Running of homozygosity (ROH) and linkage disequilibrium (LD) decay.** (A) The total ROH values of each individual in four Chinese geographical origin populations and European domestic pigs. (B) LD decay of four Chinese geographical origin populations and European domestic pigs. Abbreviations for populations and their geographic groups are given in Supplementary Table S1.

**
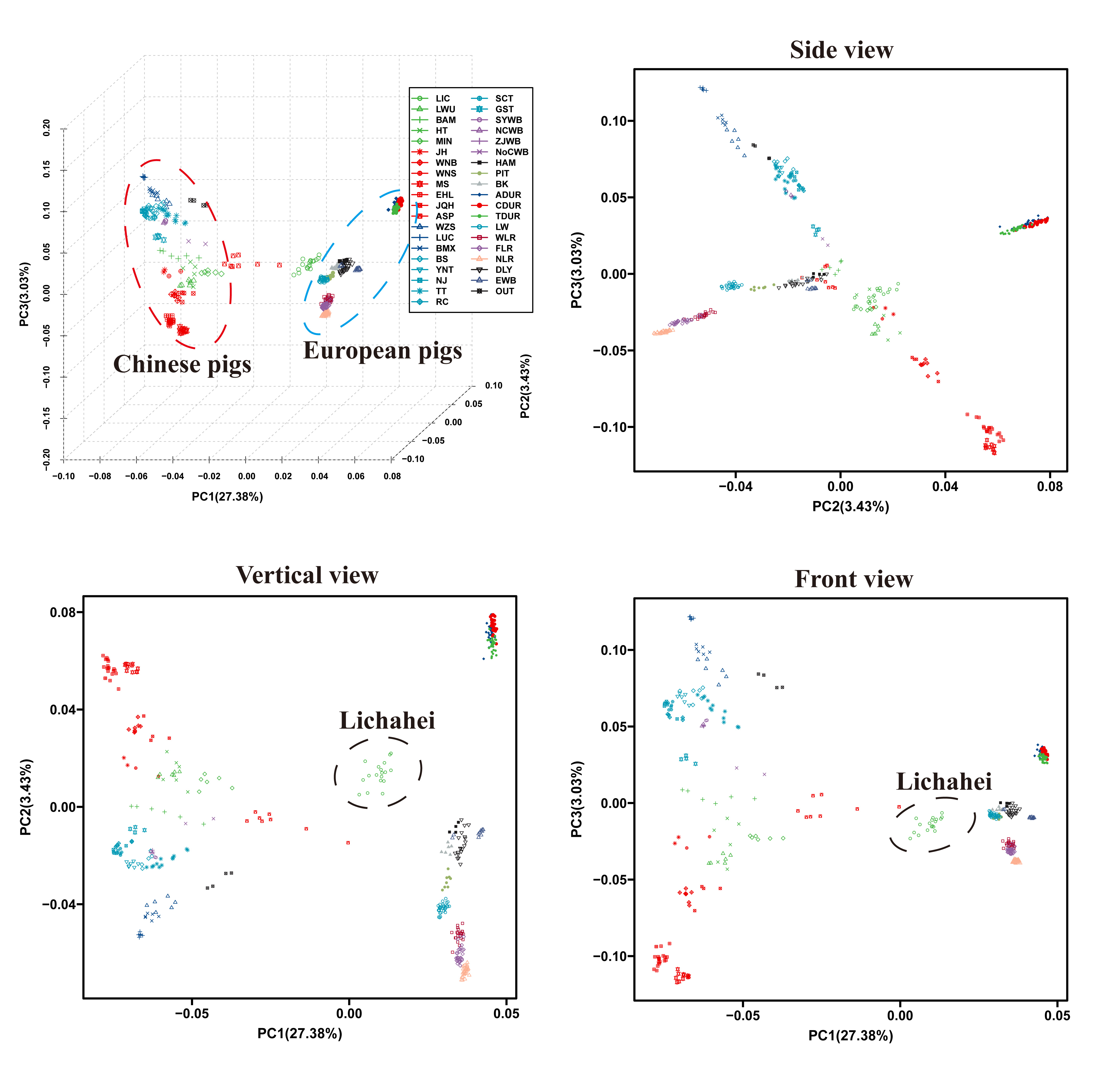
**

**Figure S4. Three-dimensional principal component analysis and** **principal component tri-views of Chinese and European pigs**. Abbreviations for populations and their geographic groups are given in Supplementary Table S1.


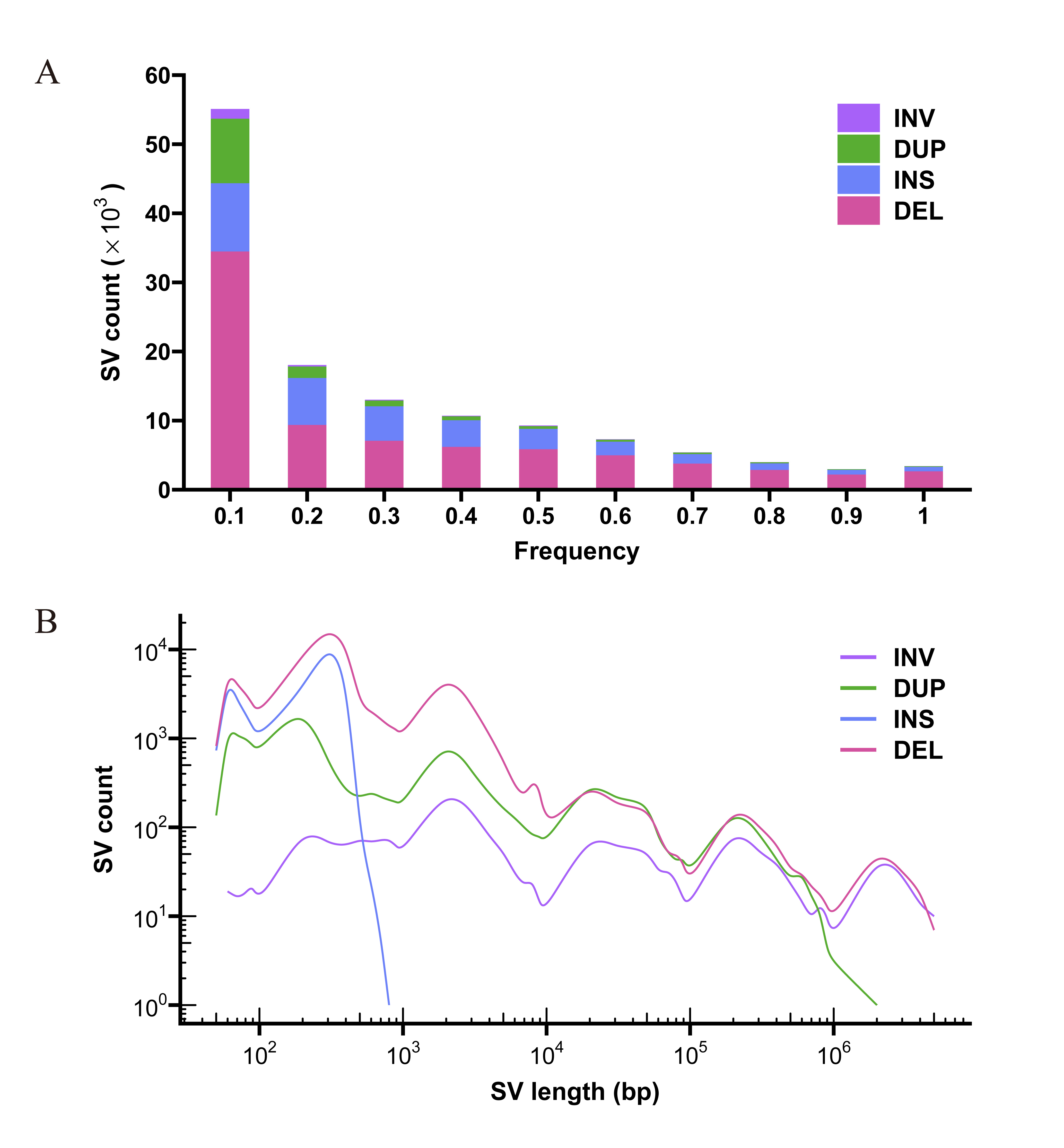


**Figure S5. Allele frequency and length distribution of the SVs of each class from 330 samples**. (A) Allele frequency of SVs from 330 samples. (B) Length distribution of the SVs of each class.


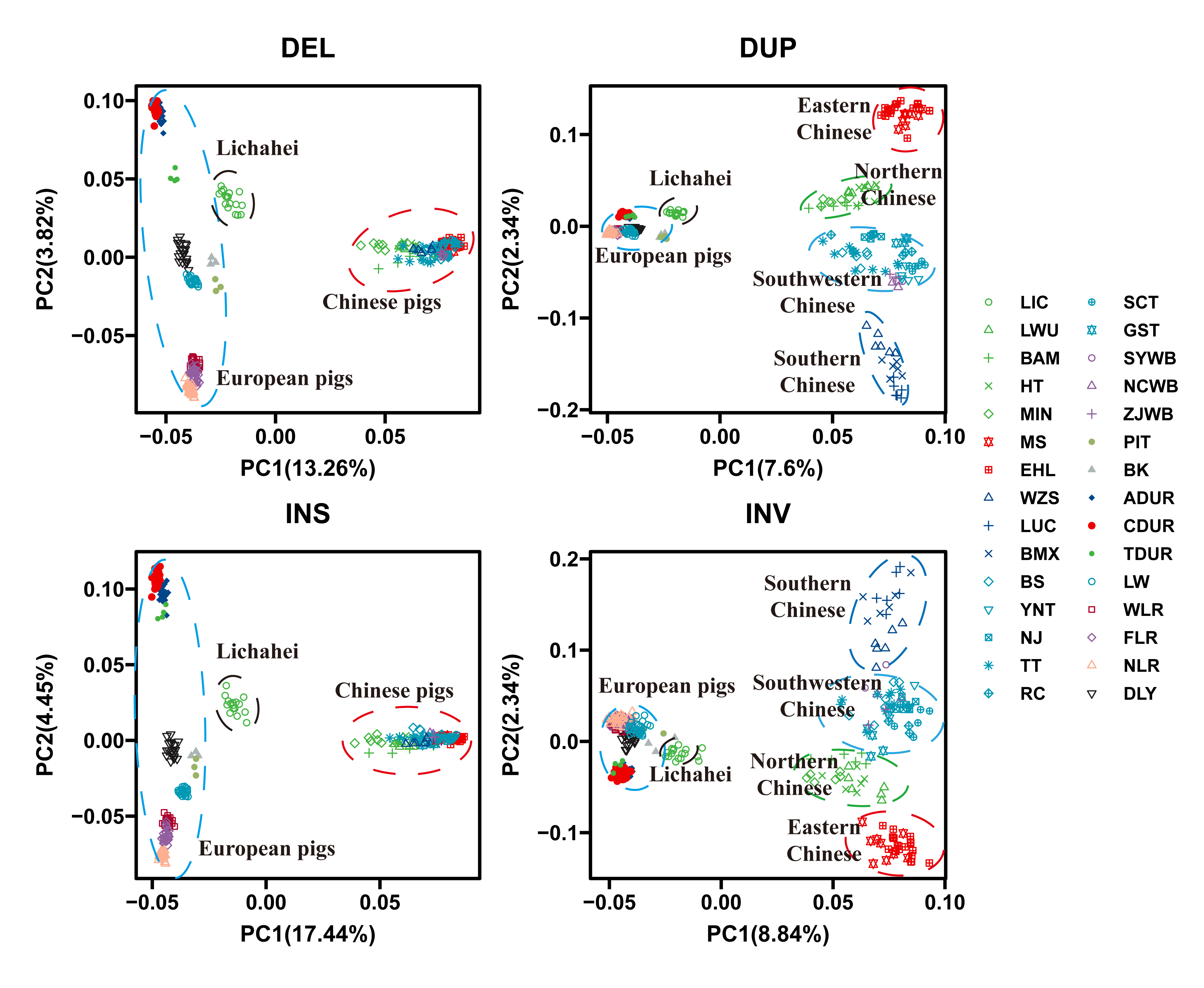


**Figure S6. PCA structure for each class of SV.** The color and shape of each point represent the breeds and geographic groups information per sample. Abbreviations for each population are given in Supplementary Table S1.


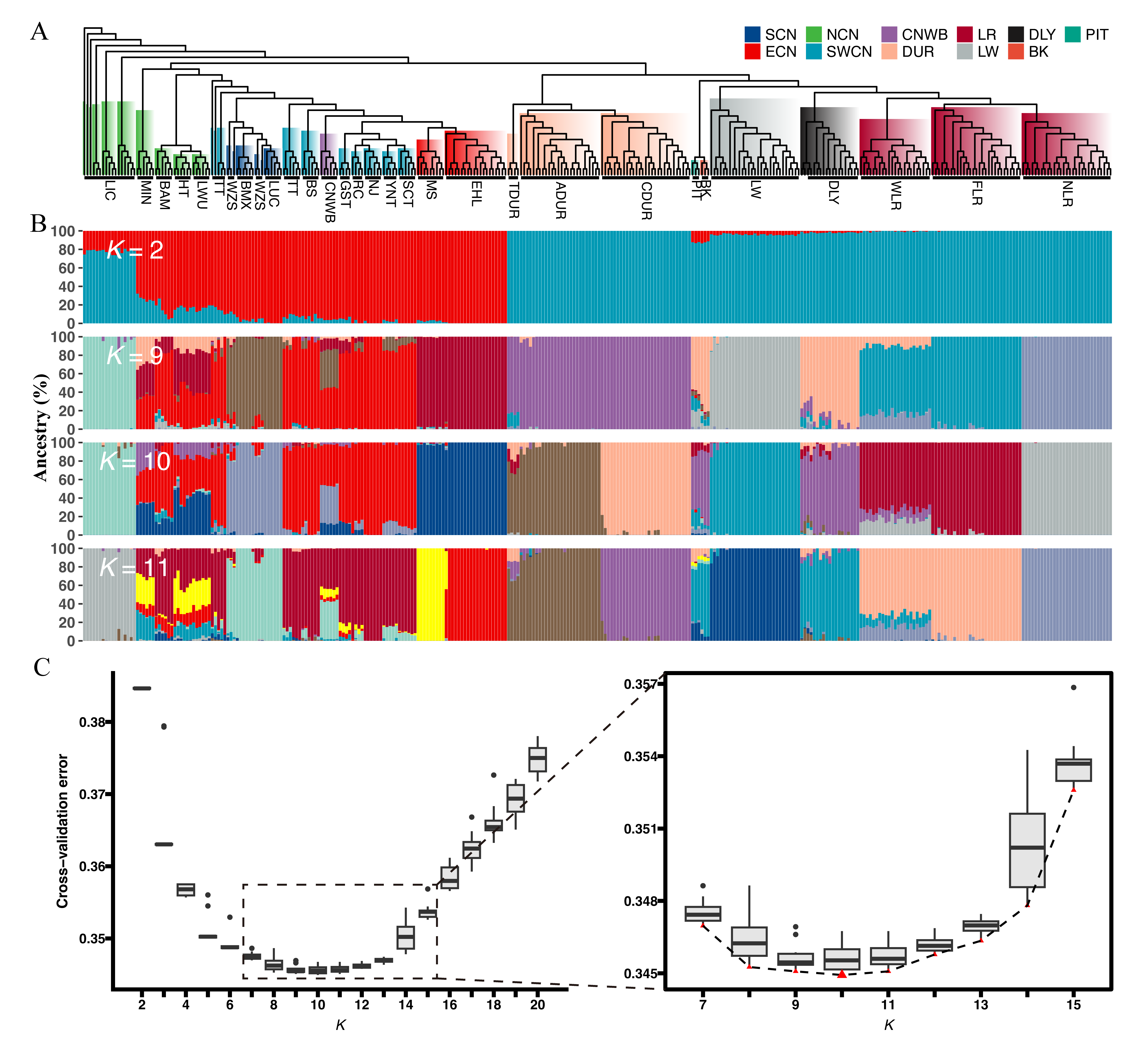


**Figure S7. Phylogenetic relationships and population structure of Chinese and European pigs based on SV.** (A) A neighbor-joining tree based on SVs. Branch colors denote populations and geographic groups. Abbreviations for each population are given in Supplementary Table S1. (B) Admixture analysis based on SVs. (C) Cross-validation errors for each *K* and those for *K* from 7 to 15 are shown in the right panel. Each box plot represents the Cross-validation errors from 10 independent runs with randomly chosen seeds.

**
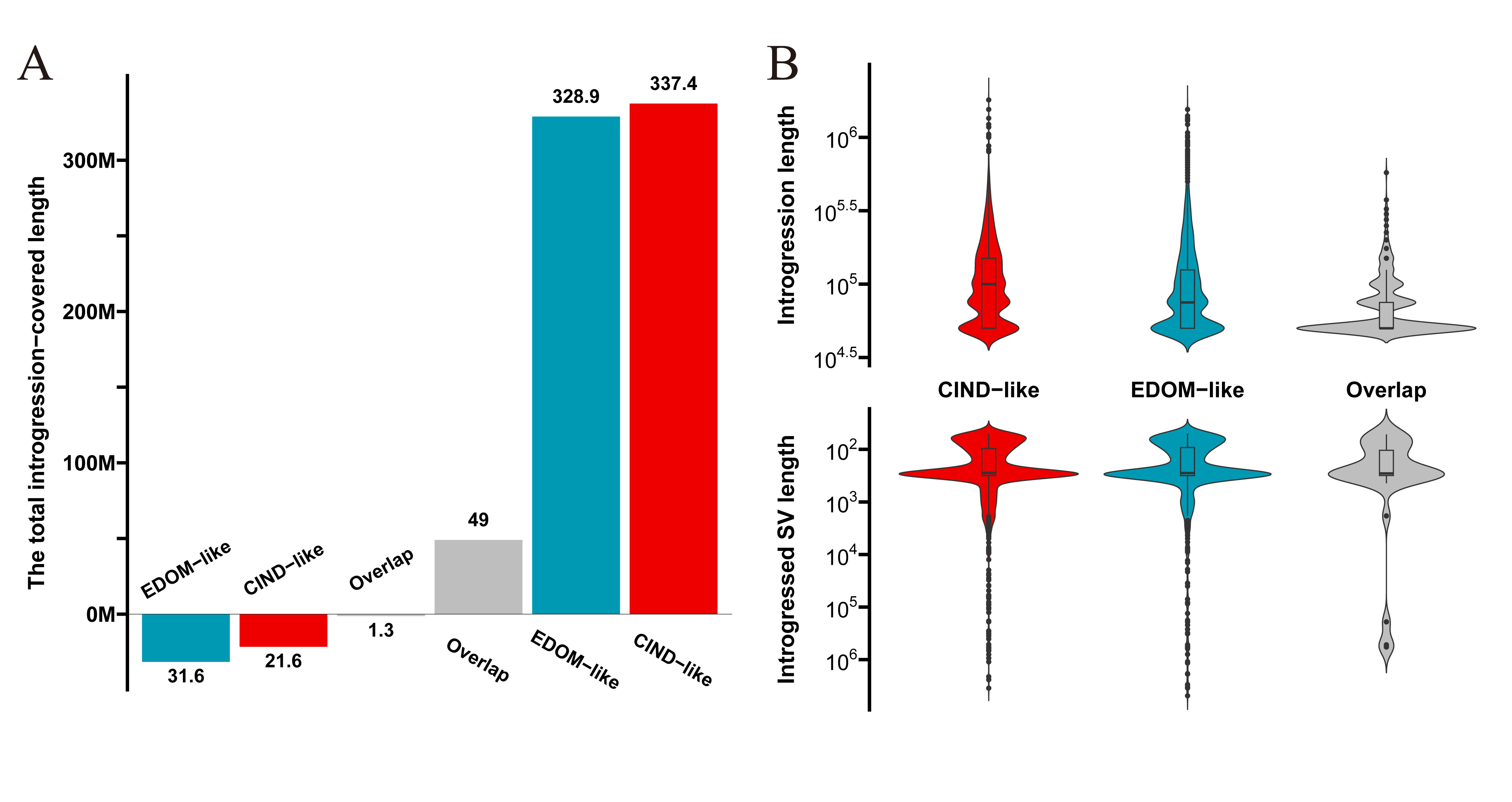
**

**Figure S8. Total length and distribution of CIND-like, EDOM-like, and candidate bi-directional (overlap) introgressed fragments and structural variations (SVs).** (A) The total introgression-covered length, with the upper half of the histogram (top) representing CIND-like, EDOM-like, and candidate bi-directional (overlap) fragments, and the lower half (bottom) representing SVs. (B) The distribution of introgressed fragment lengths, with the top panel displaying CIND-like, EDOM-like, and candidate bi-directional (overlap) fragment lengths, and the bottom panel showing the corresponding lengths of introgressed SVs.

**
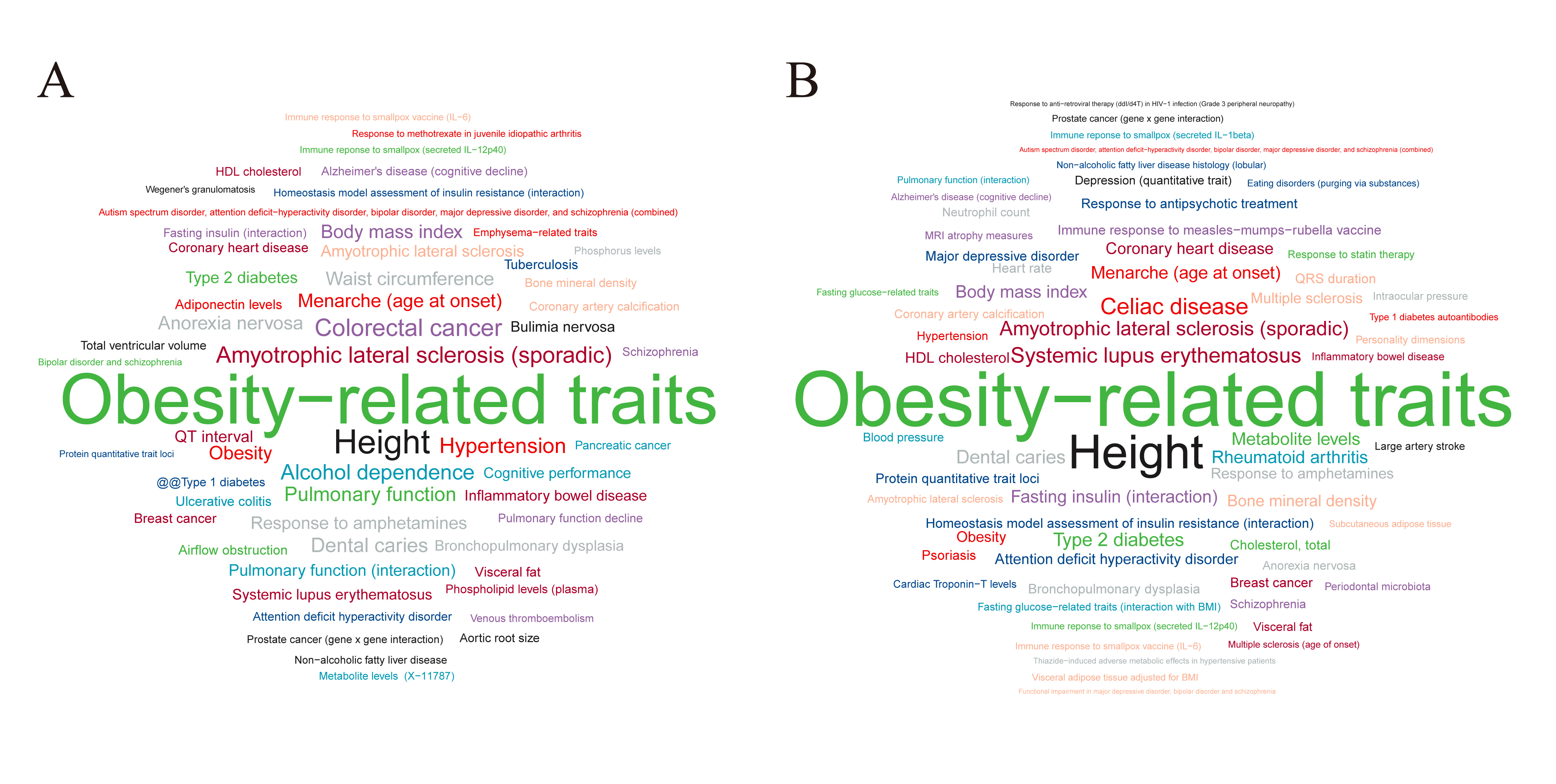
**

**Figure S9. Word cloud of the NHGRI GWAS Catalog enrichment for genes overlapping with the concatenated introgressed regions** **and those located near or overlapping SVs.** (A) Significantly enriched NHGRI GWAS Catalog of genes in the introgressed regions and SVs from Chinese indigenous pigs to European domestic pigs. (B) Significantly enriched NHGRI GWAS Catalog of genes in the introgressed regions and SVs from European domestic pigs to Chinese indigenous pigs.

**
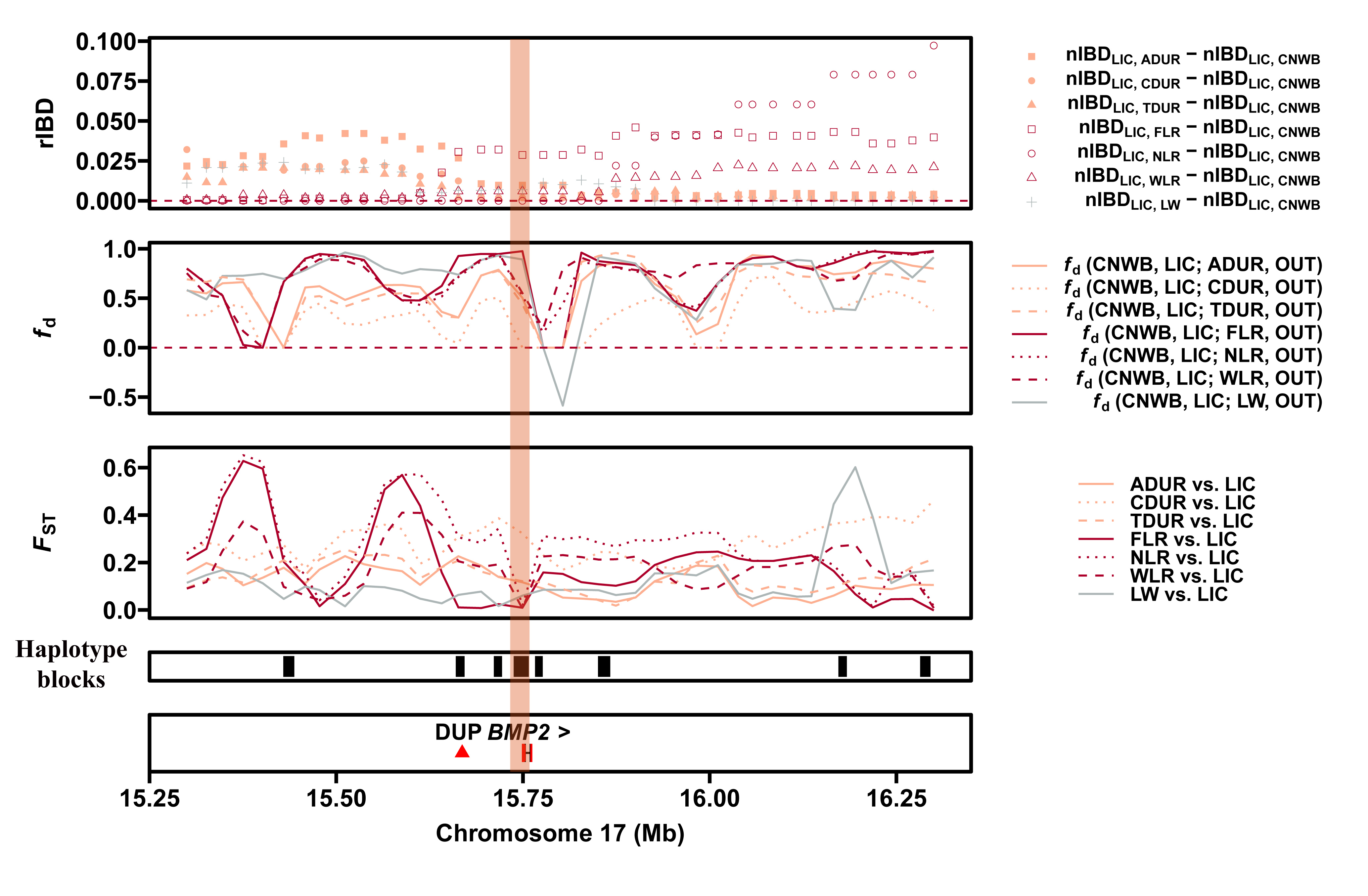
**

**Figure S10. Identification and annotation of introgression segment at the *BMP2* locus from European breeds to Lichahei pigs.** (A) Distribution of rIBD (nIBD_LIC, EDOM_ – nBID_LIC, CNWB_), $f_{d}$ (((CNWB, LIC), EDOM), Warthog), *F*_ST_, and LD blocks surrounding the introgressed regions. A 15.9 kb duplication (chr17:15660659-15676598) and *BMP2* gene were annotated in the box below the plot and according to the Ensembl Release 110 annotation. Abbreviations for each population are given in Supplementary Table S1.

**
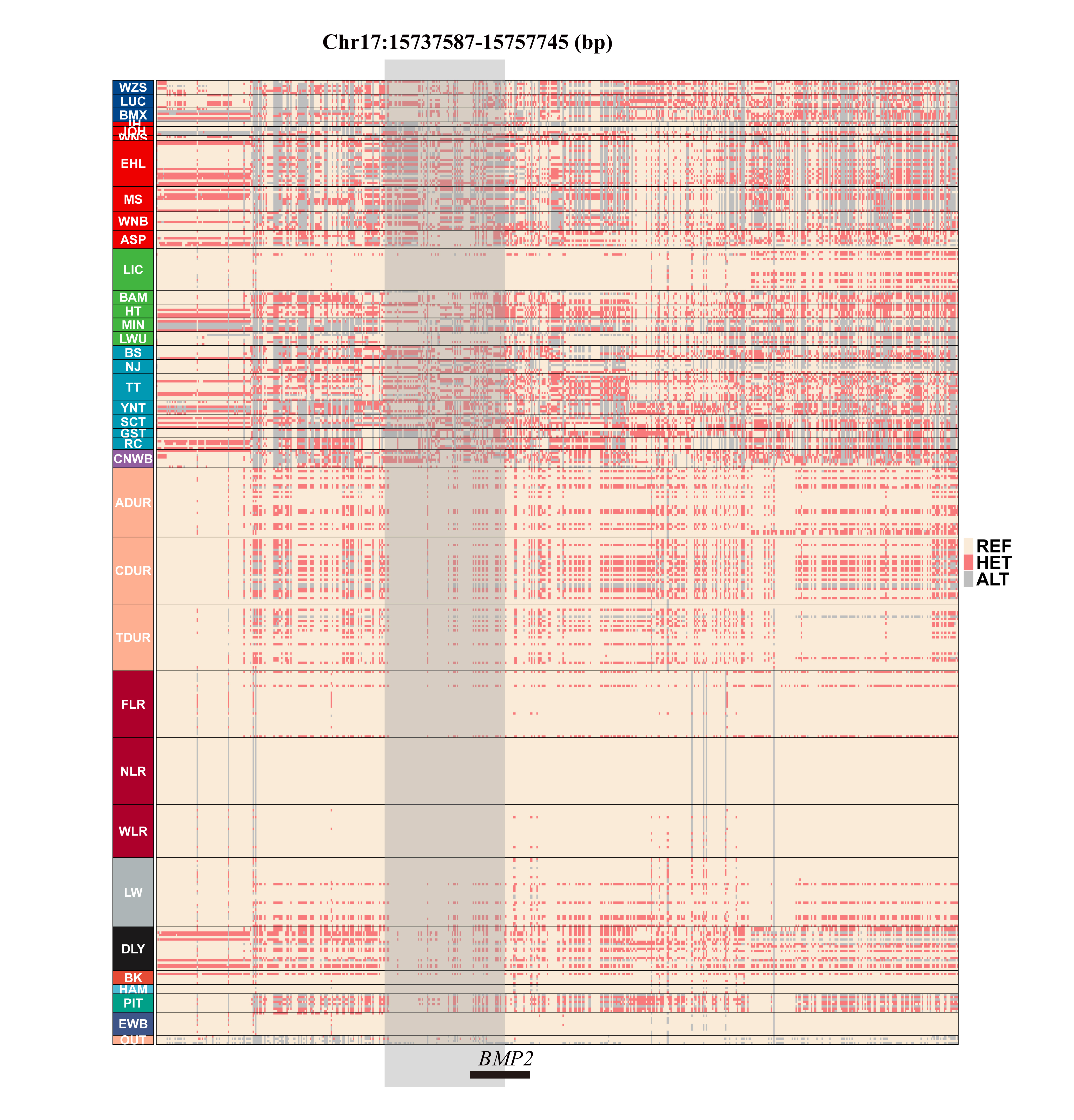
**

**Figure S1****1. Genotype patterns of phased SNPs within a 50 kb flanking region around the *BMP2* gene in 418 pigs.** Each column represents a SNP variant, and each row represents an individual. The *BMP2* haplotype (chr17:15,737,587-15,757,745) is indicated by the grey shaded box. Abbreviations for each population are given in Supplementary Table S1.


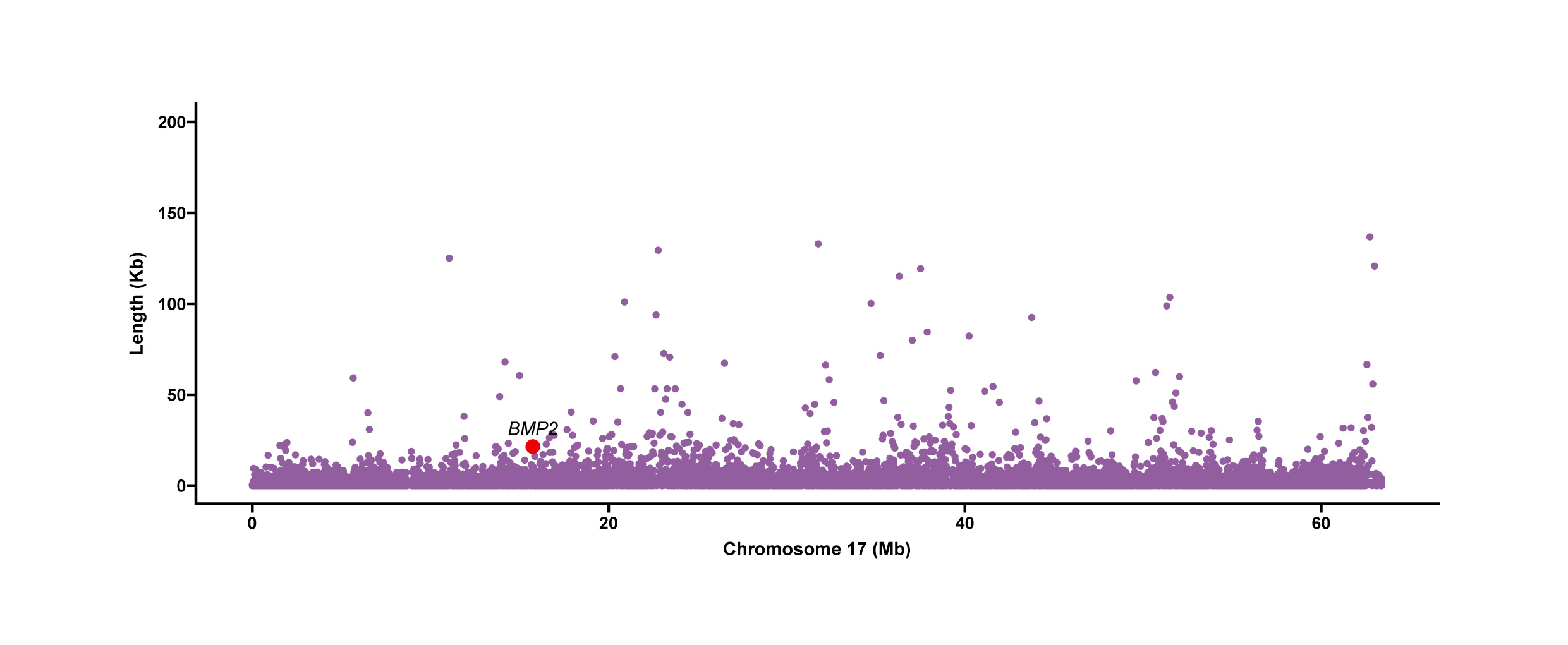


**Figure S12. The length distribution of LD blocks on chromosomes 17.** Red dots indicate the blocks located in introgressed segments of *BMP2*.


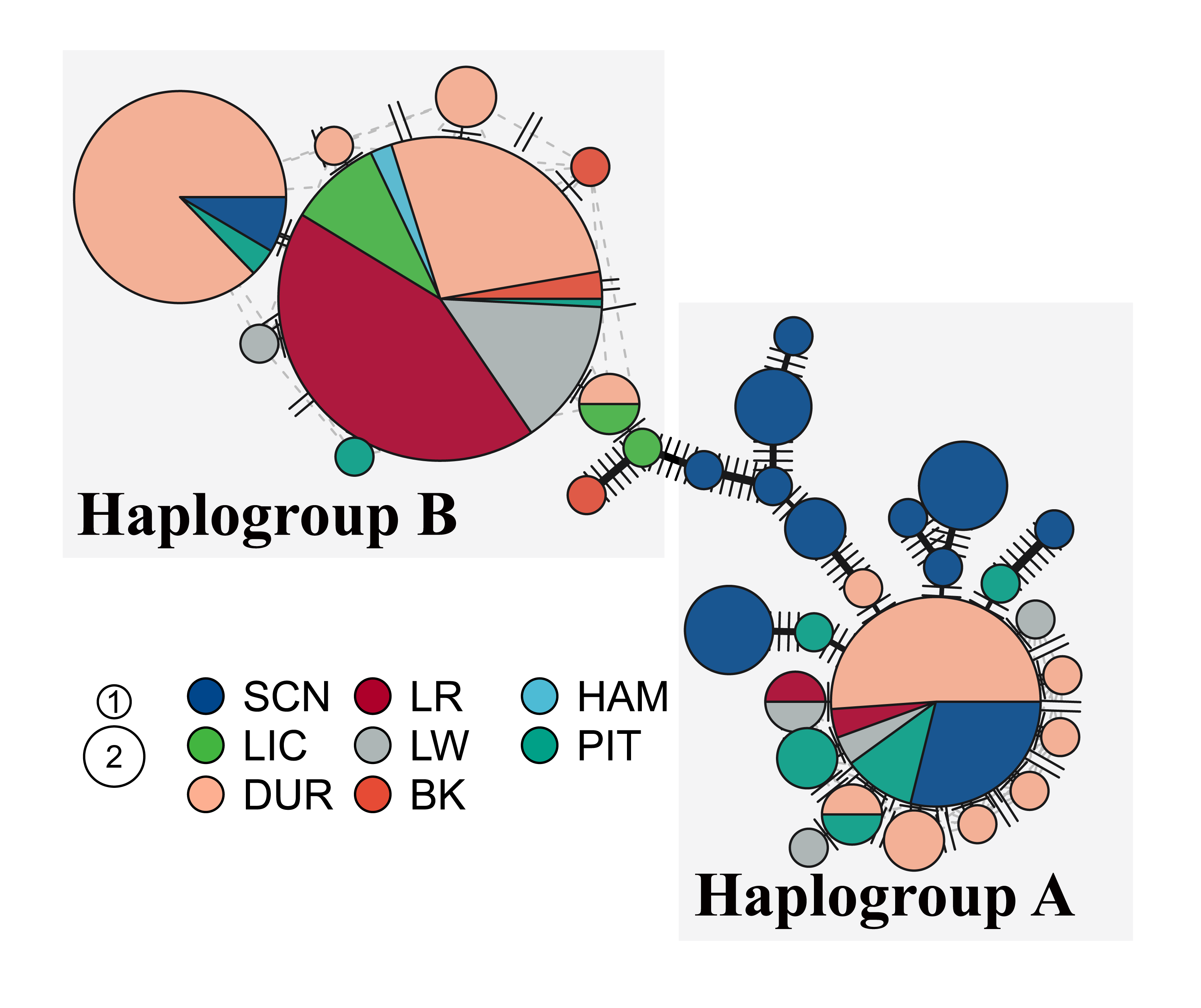


**Figure S13. A haplotype network generated based on 22 SNPs in *BMP2* haplotype (chr17:15,737,587-15,757,745).** Abbreviations for each population are given in Supplementary Table S1.


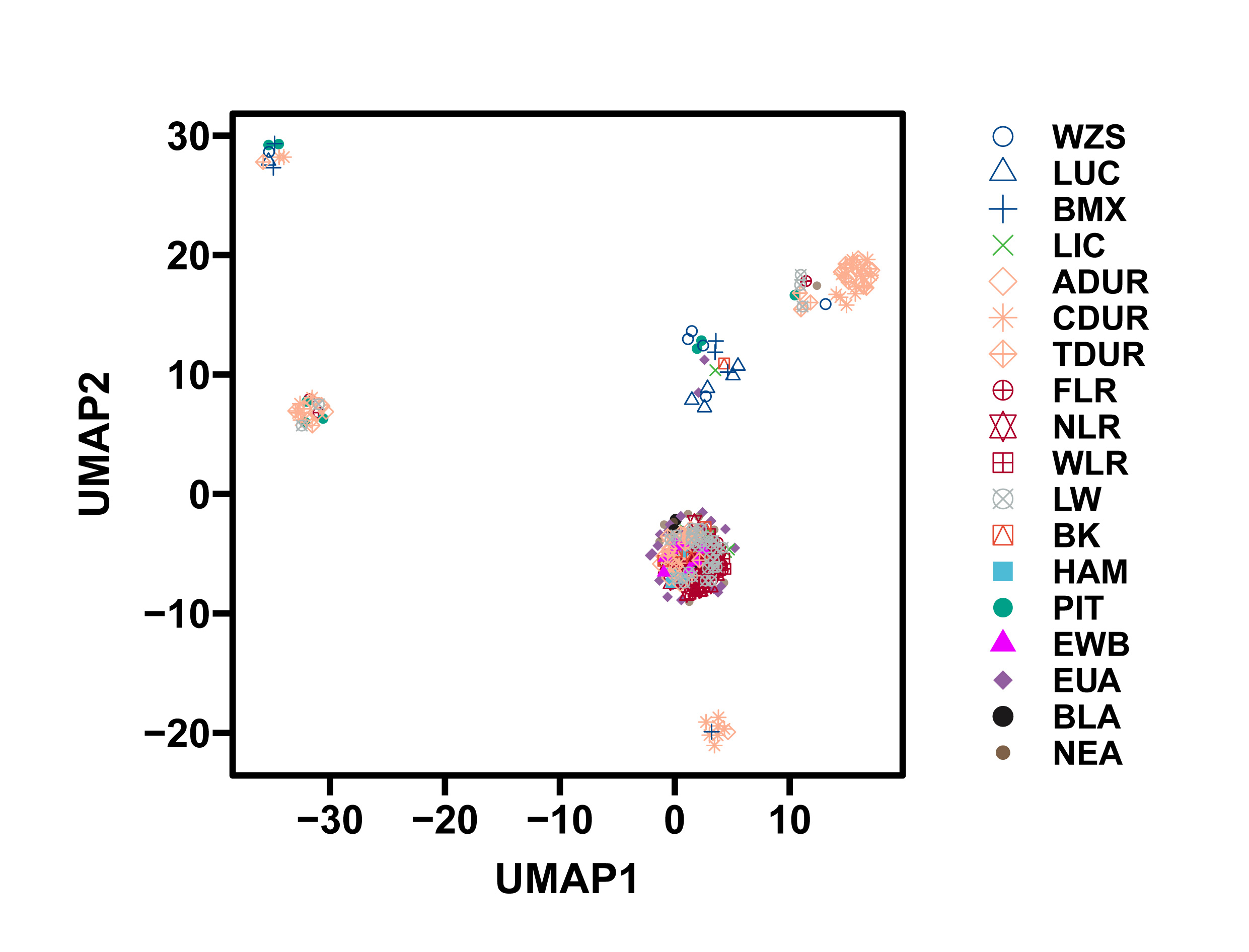


**Figure S14. UMAP of the 20.2 kb LD blocks around the *BMP2* gene.** Abbreviations for each population are given in Supplementary Table S1.


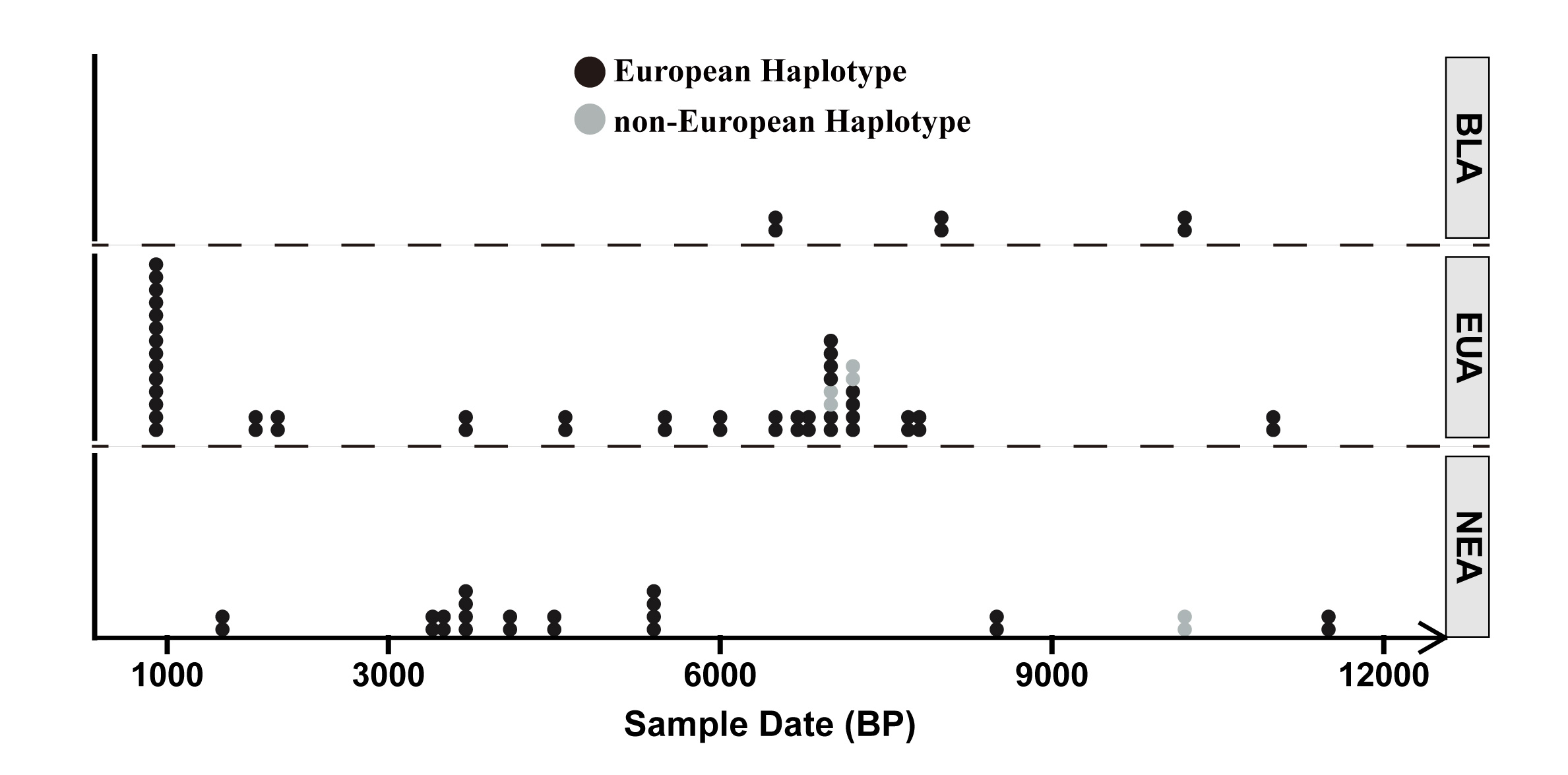


**Figure S15. The European/Near Eastern *BMP2* haplotype counts through time.** Abbreviations for each population are given in Supplementary Table S1.


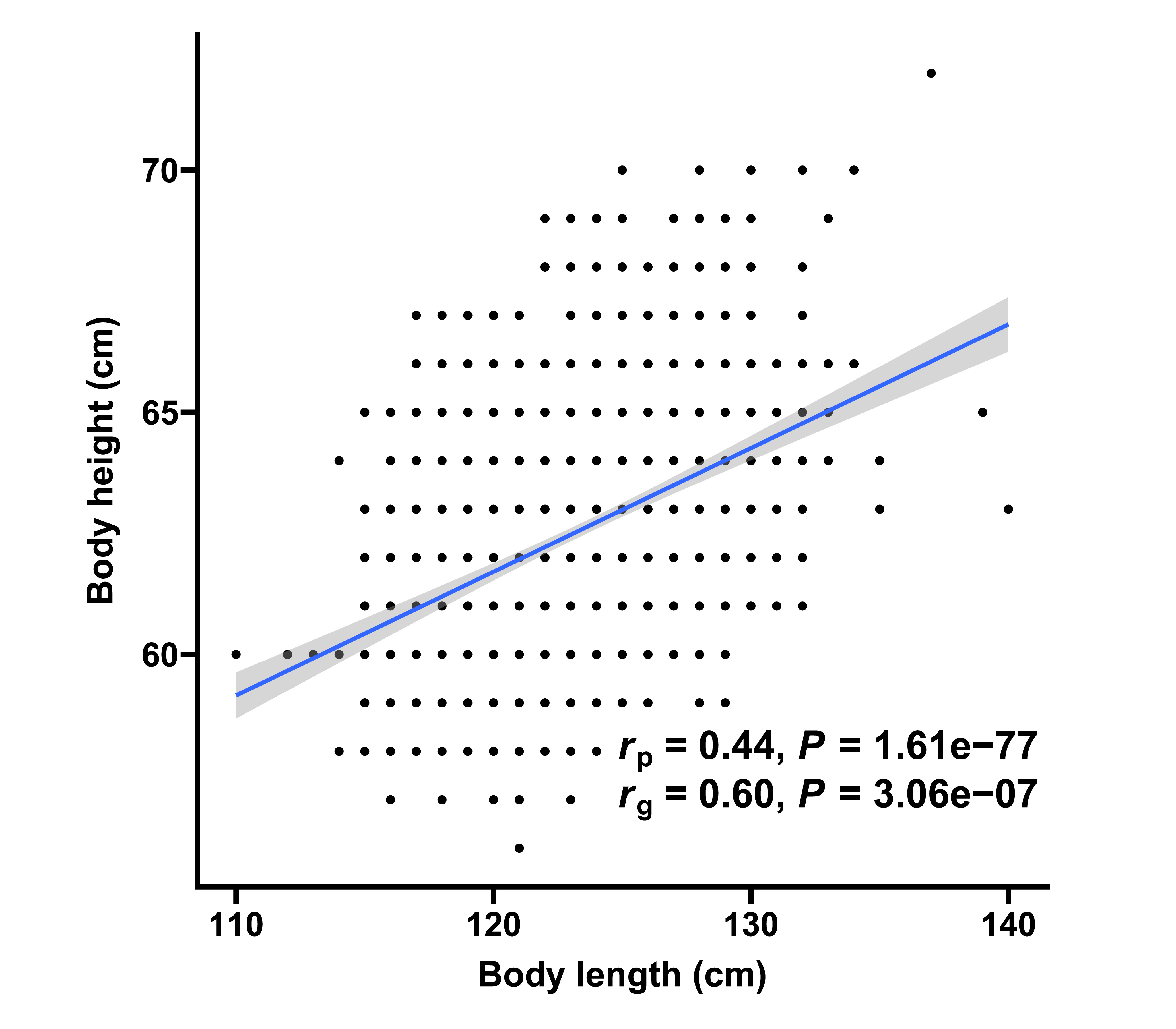


**Figure S16. Correlation analysis between body length and body height in 1,618 French Large White pigs.** Genetic and Pearson’s correlation coefficient with their *P*-values are shown at the right bottom of the plot.


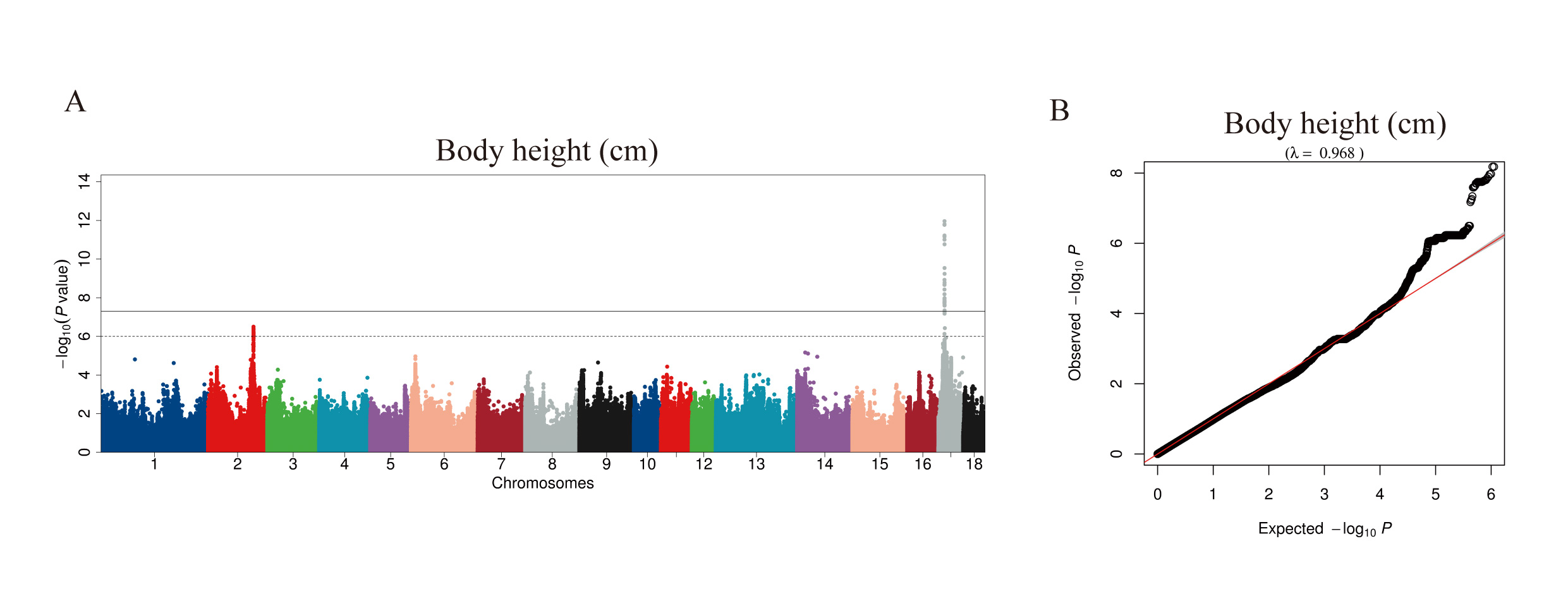


**Figure S17. Genome-wide association analysis of body height using the 1,618 French Large White pigs.** (A) Manhattan plots of body height. The x-axis represents the chromosomes, and the y-axis represents the −log10(*P*-value). The solid and dashed lines indicate the genome-wide (5 × 10^−8^) and suggestive (1 × 10^−6^) significance thresholds, respectively. (B) Quantile-quantile (Q-Q) plots show the observed versus expected negative log 10 *P*-values.


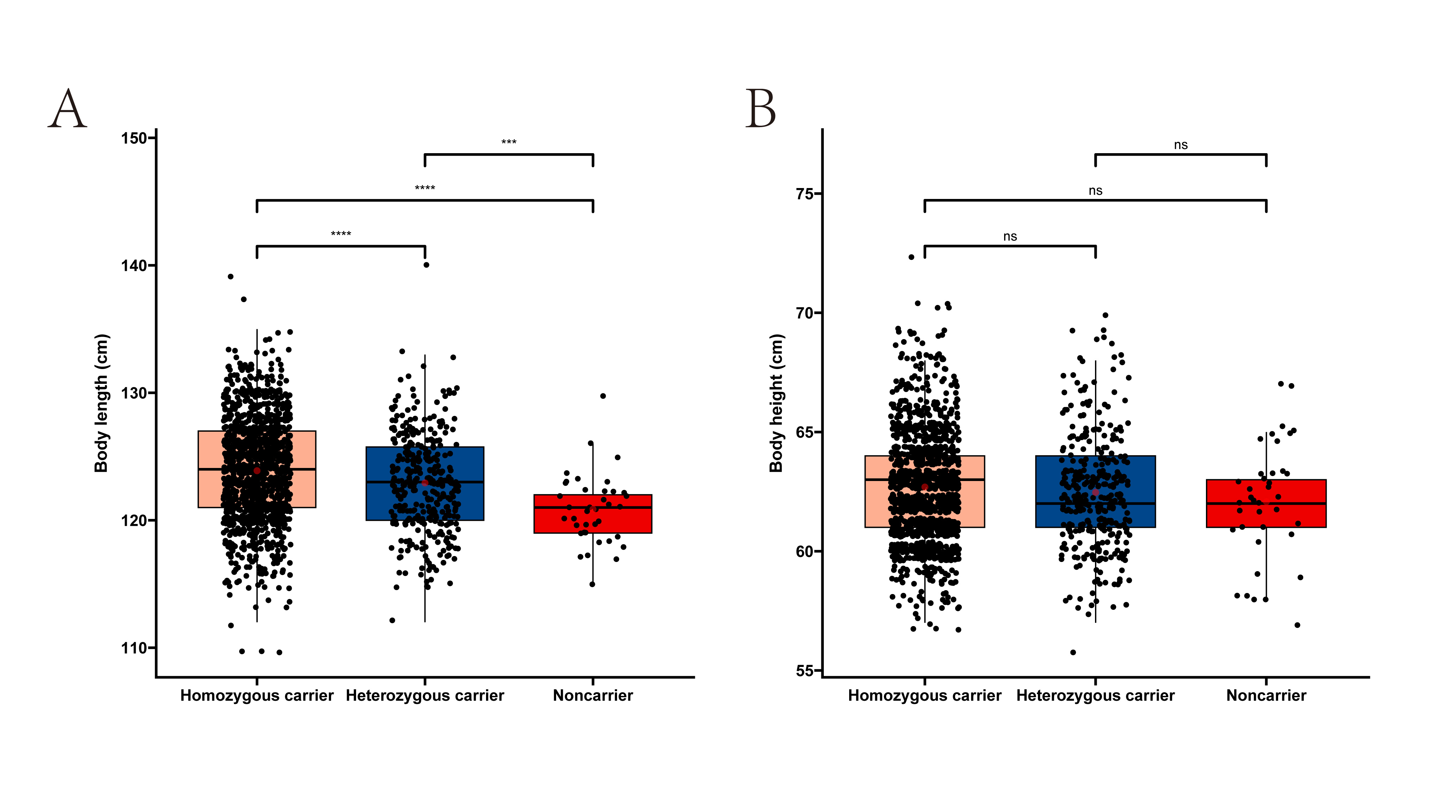


**Figure S18. Association analysis of body length and body height of *BMP2* haplotype using the 1,618 French Large White pigs.** (A) Body length. (B) Body height. The Wilcoxon rank sum test was used to compute the *P*-value.


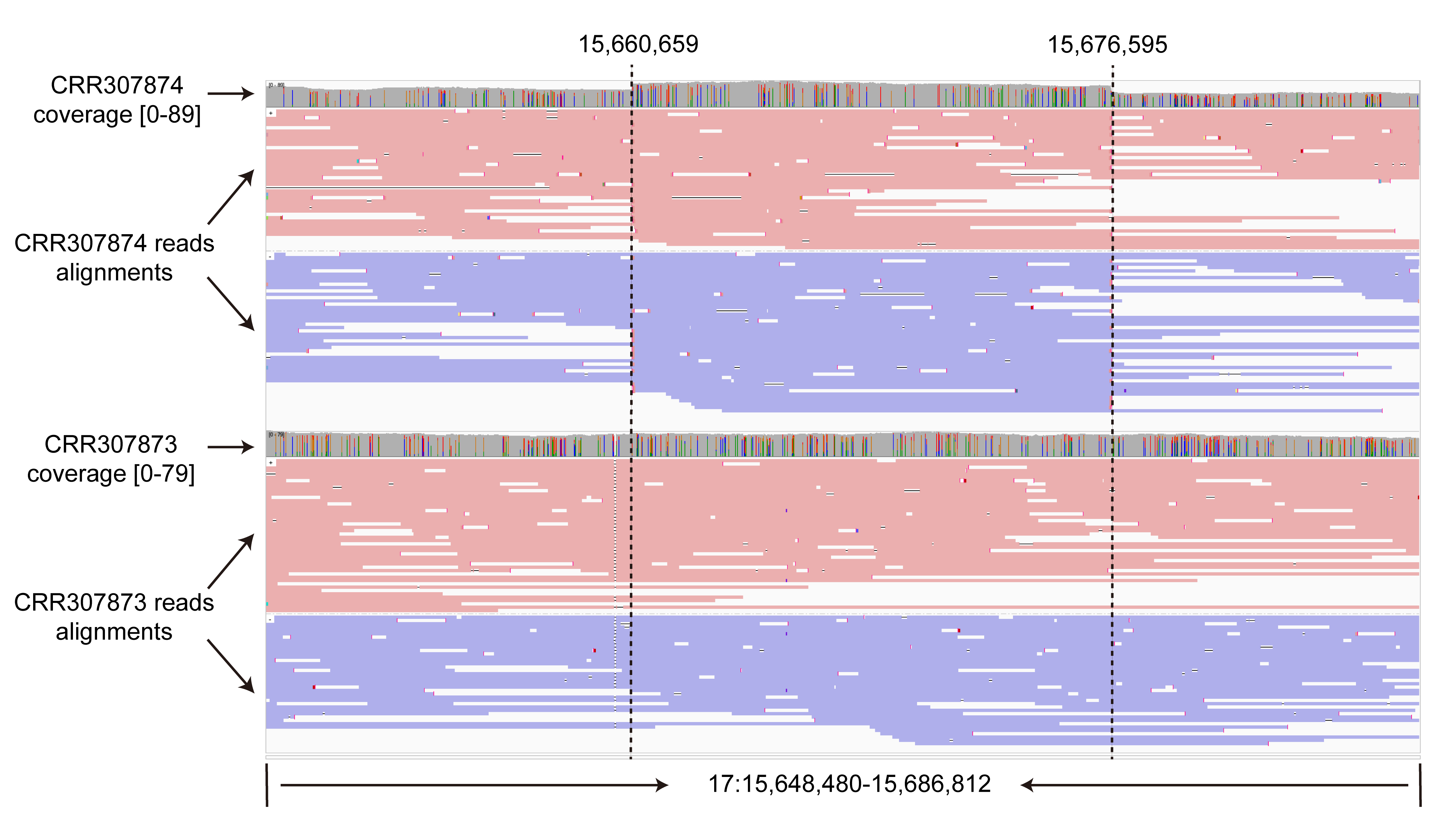


**Figure S19. IGV screenshot of the 15.9 kb duplication validated by long-read sequencing data.** The genotype of CRR307874 in this duplication region is shown as 0/1, while the genotype of CRR307873 is shown as 0/0.

**
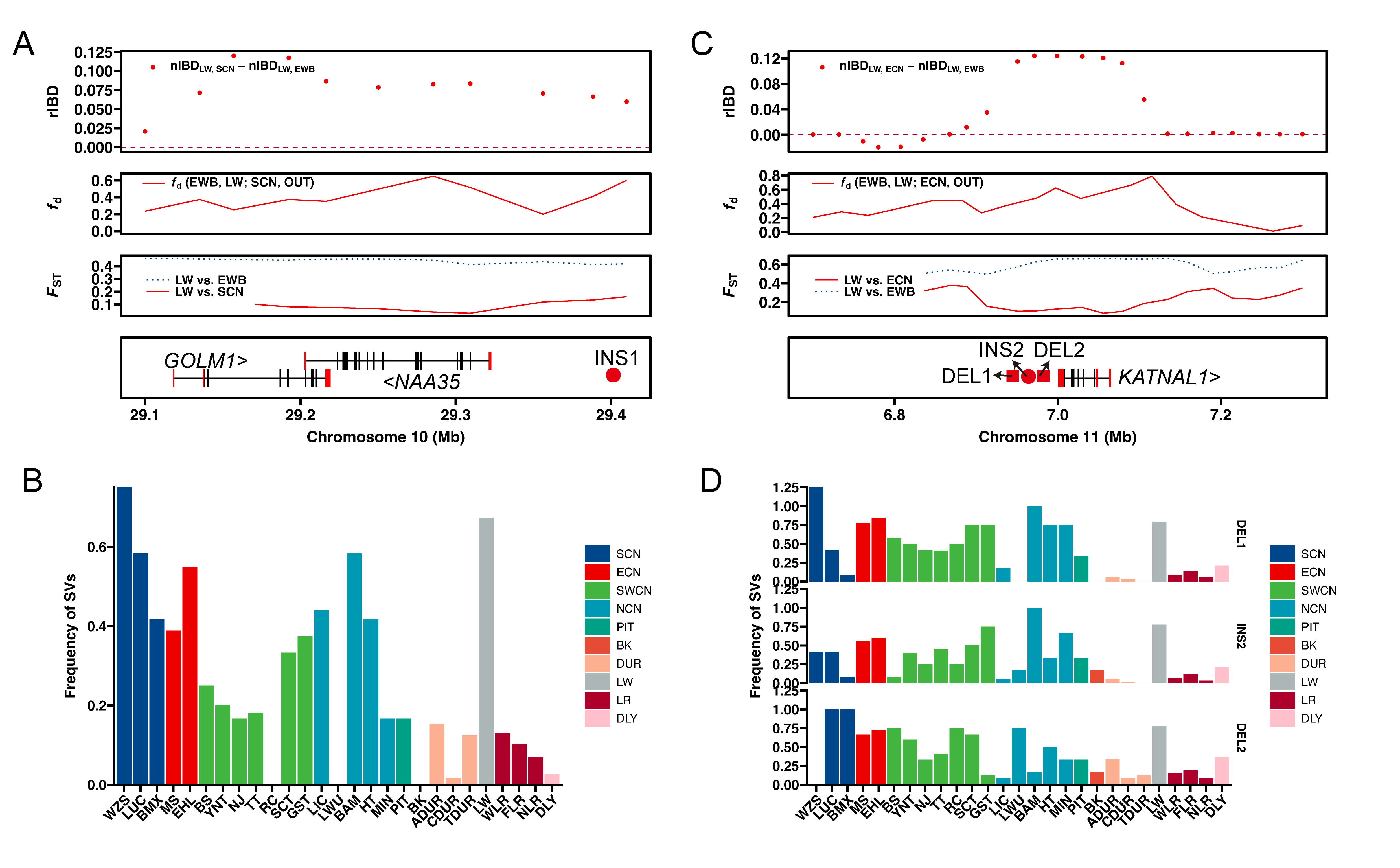
**

**Figure S20. Introgression at *GOLM1*-*NAA35*, and *KATNAL1* locus.** (A) Distribution of rIBD (nIBD_EDOM, SCN_ – nIBD_LW, EWB_), $f_{d}$ (((EWB, LW), SCN), Warthog), and *F*_ST_ surrounding the introgressed regions, respectively. (B) The frequency of the 308 bp insertion (INS1; chr10: 29,400,717) in each pig group. (C) Distribution of rIBD (nIBD_EDOM, ECN_ – nIBD_LW, EWB_), $f_{d}$ (((EWB, LW), ECN), Warthog), and *F*_ST_ surrounding the introgressed regions, respectively. (D) The frequency of the 357 bp deletion (DEL1; chr11: 6,960,754- 6,961,111), 51 bp insertion (INS2; chr11: 6,962,471), and 314 bp deletion (DEL2; chr11: 6,970,428- 6,970,742) in each pig group.

**Legends for Supplementary Table S1 to S26：**

**Supplementary Table S1 (separate file).** Summary of individual information in this study.

**Supplementary Table S2 (separate file).** Samples and their genomic diversity statistics.

**Supplementary Table S3 (separate file).** Weighted *F*_ST_ between each population.

**Supplementary Table S4 (separate file).** The *D* and related statistics across the combinations of trio population.

**Supplementary Table S5 (separate file).** The landscape of SVs between CIND pigs and EDOM pigs.

**Supplementary Table S6 (separate file).** Simulations of the increase of the pan-SV size and the decrease of core-SV size.

**Supplementary Table S7 (separate file).** The distribution of SVs between CIND pigs and EDOM pigs.

**Supplementary Table S8 (separate file).** The 50 kb regions of potential introgression from Chinese indigenous pigs into European domestic pigs (CIND-like).

**Supplementary Table S9 (separate file).** The 50 kb regions of potential introgression from European domestic pigs into Chinese indigenous pigs (EDOM-like).

**Supplementary Table S10 (separate file).** The frequencies of introgressed SVs from Chinese indigenous pigs into European domestic pigs (CIND-like).

**Supplementary Table S11 (separate file).** The frequencies of introgressed SVs from European domestic pigs into Chinese indigenous pigs (EDOM-like).

**Supplementary Table S12 (separate file).** The 50 kb regions of potential introgression showing bi-directional signals.

**Supplementary Table S13 (separate file).** The frequencies of introgressed SVs showing bi-directional signals.

**Supplementary Table S14 (separate file).** Significantly enriched NHGRI GWAS Catalog of genes overlapping the introgressed regions and those located near or overlapping SVs between Chinese indigenous pigs and European domestic pigs.

**Supplementary Table S15 (separate file).** The 50 kb regions of potential introgression from Southern Chinese indigenous pigs into European domestic pigs around the *BMP2* gene.

**Supplementary Table S16 (separate file).** The 50 kb regions of potential introgression from European domestic pigs into Lichahei pigs around the *BMP2* gene.

**Supplementary Table S17 (separate file).** Numbers of different haplotypes of introgressed region in *BMP2* gene among Southern Chinese indigenous, Lichahei, and European domestic pig populations.

**Supplementary Table S18 (separate file).** European haplotype of introgressed region in *BMP2* gene among modern and ancient pigs.

**Supplementary Table S19 (separate file).** GWAS significant SNPs (*P* < 5 x 10^-8^) for body height using imputed genotypes.

**Supplementary Table S20 (separate file).** *BMP2* haplotype genotype, body length and body height in 1,618 French Large White boars.

**Supplementary Table S21 (separate file).** Allele frequencies of SNP and SVs among Chinese and European pigs.

**Supplementary Table S22 (separate file).** The linkage disequilibrium between the significant SNP (chr17:15,643,342:C>T) and SVs within a 1 Mb range both upstream and downstream.

**Supplementary Table S23 (separate file).** Genotype of the 15.9 kb duplication (chr17:15,660,659-15,676,598) in the 10 publicly available pig genomes.

**Supplementary Table S24 (separate file).** The Chromatin state overlapped with SVs.

**Supplementary Table S25 (separate file).** Admixture analysis based on SNPs (ten random seeds were run for each *K*).

**Supplementary Table S26 (separate file).** Admixture analysis based on SVs (ten random seeds were run for each *K*).
